# Decoding the metabolic response of *Escherichia coli* for sensing trace heavy metals in water

**DOI:** 10.1073/pnas.2210061120

**Published:** 2023-02-06

**Authors:** Hong Wei, Yixin Huang, Peter J. Santiago, Khachik E. Labachyan, Sasha Ronaghi, Martin Paul Banda Magana, Yen-Hsiang Huang, Sunny C. Jiang, Allon I. Hochbaum, Regina Ragan

**Affiliations:** ^a^Department of Materials Science and Engineering, University of California, Irvine, CA 92697-2585; ^b^Department of Chemical and Biomolecular Engineering, University of California, Irvine, CA 92697-2580; ^c^Department of Pharmaceutical Sciences, University of California, Irvine, CA 92697-3958; ^d^Sage Hill School, Newport Coast, CA 92657; ^e^Department of Molecular Biology and Biochemistry, University of California, Irvine, CA 92697-2525; ^f^Department of Civil and Environmental Engineering, University of California, Irvine, CA 92697-2175; ^g^Department of Ecology and Evolutionary Biology, University of California, Irvine, CA 92697-2525; ^h^Department of Chemistry, University of California, Irvine, CA 92697-2025

**Keywords:** bacterial metabolism, machine learning, vibrational spectroscopy, environmental sensors

## Abstract

Heavy metal contamination due to industrial and agricultural waste represents a growing threat to water supplies. Frequent and widespread monitoring for toxic metals in drinking and agricultural water sources is necessary to prevent their accumulation in humans, plants, and animals, which results in disease and environmental damage. Here, the metabolic stress response of bacteria is used to report the presence of heavy metal ions in water by transducing ions into chemical signals that can be fingerprinted using machine learning analysis of vibrational spectra. Surface-enhanced Raman scattering surfaces amplify chemical signals from bacterial lysate and rapidly generate large, reproducible datasets needed for machine learning algorithms to decode the complex spectral data. Classification and regression algorithms achieve limits of detection of 0.5 pM for As^3+^ and 6.8 pM for Cr^6+^, 100,000 times lower than the World Health Organization recommended limits, and accurately quantify concentrations of analytes across six orders of magnitude, enabling early warning of rising contaminant levels. Trained algorithms are generalizable across water samples with different impurities; water quality of tap water and wastewater was evaluated with 92% accuracy.

## Significance

The biochemical network stress response of *Escherichia coli* reports the presence of heavy metal contaminants in water when integrated with optical sensors. Machine learning analysis of the vibrational spectra of metabolites released in response to chromium and arsenic exposure detects concentrations 10^8^ times lower than those leading to cell death. Heavy metal type and concentration are determined with accuracy exceeding 92%, which is promising for longitudinally monitoring changes in water quality. Transfer learning of trained algorithms is further demonstrated to be generalizable to unseen tap water and wastewater samples where data acquisition requires less than 10 min for evaluation of water quality.

Like all living organisms, bacteria are equipped with biochemical machinery to survive and adapt in diverse and changing environments all over the world. These responses to dynamic conditions elicit changes in bacteria metabolic networks, and their metabolite profiles can shift on timescales as short as minutes (1). Many of these environmental changes constitute stresses, which trigger physiological responses within the cell. Stresses, ranging from nutrient restriction (2) to exposure to antibiotics (3), elicit profound metabolic consequences in bacteria. The resulting changes in metabolite profiles can be detected by conventional (3) and next-generation (4) metabolomic techniques. Consequently, we hypothesize and demonstrate that bacterial cultures can be used as whole-cell sensors of environmental stressors by the detection and decoding of their metabolic responses to these stressors. Specifically, the bacterial metabolic response transduces heavy metal ions in water into chemical (metabolite) signals that are amplified with surface-enhanced Raman scattering (SERS) surfaces. When decoding the spectral signals using machine learning (ML) algorithms, a sensitive and accurate sensing platform for ensuring water safety results.

Heavy metal contamination from natural and anthropogenic sources is a serious threat to human and ecosystem health, and heavy metal use in a wide variety of industrial and agricultural processes is growing exponentially (5, 6). Contaminated water is a major source of exposure leading to toxic heavy metal accumulation in humans, plants, and livestock. The development of portable and low-cost sensors which can be broadly deployed to locally and frequently monitor the quality of drinking and irrigation water, agricultural, and industrial runoff is needed to safeguard sensitive ecosystems and human health. Arsenic, cadmium, chromium, copper, lead, and mercury rank among the priority metals of public health significance (5). Currently, monitoring water quality typically requires samples to be sent to specifically certified laboratories for inductively coupled plasma-mass spectrometry analysis for quantification (7) to determine if contaminants are below safety guidelines set by the World Health Organization (WHO) (8) or regulatory agencies. Other laboratory methods with the necessary limit of detection (LOD) and dynamic range rely on similarly sophisticated and centralized analytical instruments, such as atomic absorption, X-ray fluorescence, or atomic emission spectrometries (7).

Alternatively, biosensors, using physicochemical signal transduction, such as optical, electrochemical, piezoelectric, and thermal signal outputs, represent low-cost solutions that are compatible for integration in portable systems to detect heavy metal ions. Molecular recognition labels include enzymes (9), antibodies (10), whole cells (11), aptamers (12), molecularly imprinted polymers (13, 14), and DNA (15). Encapsulation of enzymes in hydrogels yields sensors with a LOD needed for monitoring water quality, but they have limited shelf life (9). Aptamers, on the other hand, exhibit high specificity and stability but are not easily engineered to detect a variety of analytes. Antibodies, relying on the formation of metal-chelated complexes, are versatile sensing elements, yet cross-reactivity with other ions leads to lack of specificity (16). Whole cell-based biosensors rely on mature cell culturing technology and can be incorporated in a range of physicochemical sensor platforms for multiple assays. Whole-cell biosensors have received increasing attention as an ultrasensitive means of detecting hazardous contaminants as they can be engineered to be responsive to different toxins (17).

Many cellular metabolites have high Raman cross-sections (18), which can be detected in SERS measurements (4, 19). SERS is a highly sensitive and label-free detection scheme (20), which offers single molecule LOD when using carefully designed nanoarchitectures (21–23). Indeed, SERS signals from Au-decorated nanofiber probes inserted into breast cancer cells have been shown to detect toxic metal exposure at a LOD of 5 nM for mercury and 100 nM for silver (24). Obtaining reproducible responses in biosensors is a longstanding challenge (25). In particular, the reproducibility of SERS surfaces depends on nanoparticle (NP) morphology, nanogap distance, and surface chemistry (26). Our previously demonstrated chemically assembled SERS surfaces composed of spherical NPs with a controlled nanogap spacing of 0.9 nm and chemistry exhibit reproducible billion-fold signal enhancements over areas of 1 cm^2^ (27). Chemical assembly of NPs with molecular control of nanogap spacing over large areas (27) allows for characterization with portable systems with large beam diameters. Comparison of spectral data from a self-assembled monolayer of benzenethiol on a chemically assembled sensor surface using a BWTek i-Raman Plus portable spectrometer and Renishaw InVia™ confocal Raman microscope demonstrates the C–H ring bending mode, with a small Raman cross-section (28, 29), is observable with both systems, and both systems have comparable signal to noise (*SI Appendix*, Fig. S1). Sensor surfaces are able to detect metabolites from bacterial communities on a time scale of minutes (4, 30) and accurately quantify analyte concentrations down to 10 fM when using ML analysis of spectral data (21). In this work, the sensitivity of the *Escherichia coli* (*E. coli*) stress response is used to transduce the signal of Cr^6+^ and As^3+^ ions into chemical signals that are detected with chemically assembled SERS surfaces. Arsenite is one of the most common toxic valence states (III) of As, and high arsenite concentrations are indicators of phytoplankton bloom, high microbial populations, and pollution from mining activity (31). Cr pollution is largely related to industrial applications in the field of energy production, manufacturing of metals and chemicals, and subsequent waste and wastewater management (32). Cr^6+^ is much more toxic than Cr^3+^ (8). A support vector machine (SVM) model achieves higher than 97% classification accuracy for decoding *E. coli* stress response to different concentrations of metal ions for concentrations as low as 68 pM for Cr^6+^ and 5 pM for As^3+^. Due to their distinct mechanisms of toxicity in bacteria, this sensing platform also distinguishes the metabolic response of As^3+^ and Cr^6+^ with high accuracy when analyzed with SVM models. In addition, convolutional neural networks (CNN) show sensitive and quantitative determination of concentrations across a dynamic range of 0.68 pM–68 µM for Cr^6+^ and 5 fM–5 mM for As^3+^, well below WHO recommended limits of 10 µg/L for As^3+^ and 50 µg/L for Cr^6+^, respectively (8). At the lowest concentrations investigated, the metabolic response is detectable when the ratio of metal ions to bacterium in solution is 0.6 for As^3+^ and 8.2 for Cr^6+^. Finally, by using a pretrained model for analysis of previously unseen tap water and wastewater samples spiked with As^3+^, SERS detection and ML analysis requires only 80 spectra per class (40 s total acquisition time) to achieve greater than 92% accuracy for classifying concentrations above or below the WHO recommended limit.

## Results

### Biochemical Signal Transduction of Metal Ions into Vibrational Spectra.

**Fig. 1.**
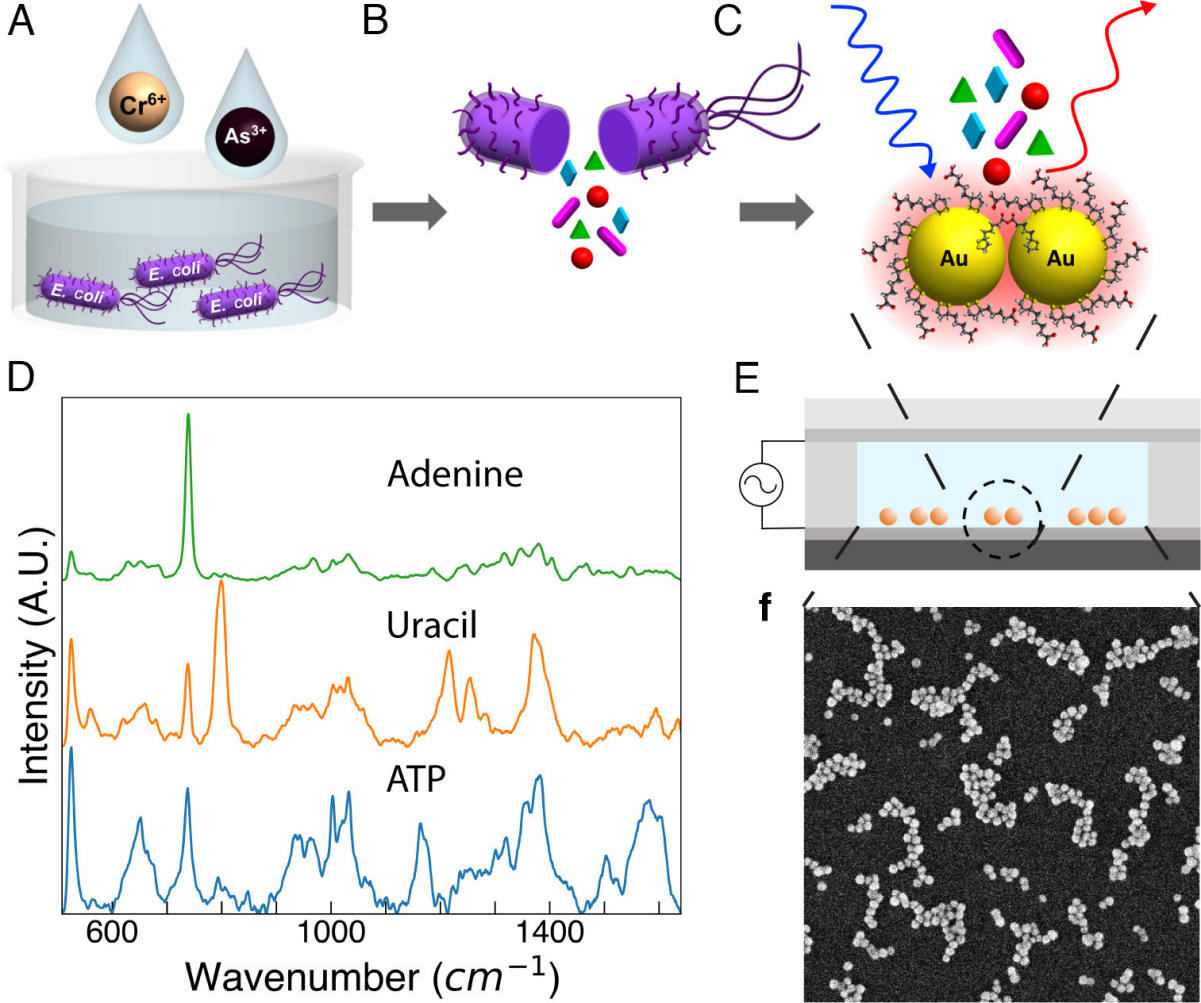
Heavy metal detection scheme and SERS spectra of key metabolites. (*A*) *E. coli* is cultured in growth media supplemented with Cr^6+^ or As^3+^ salts. (*B*) Cells are thermally lysed, and (*C*) lysate supernatant is deposited on SERS surfaces. (*D*) Representative SERS spectra of key nucleotides involved in bacterial stress responses, ATP, uracil, and adenine. (*E*) Schematic of fabrication of SERS surfaces: a microfluidic cell with an AC electric field across electrodes induces EHD flow to drive lateral assembly and subsequent cross-linking reactions between Au NP. (*F*) Scanning electron microscopy image shows Au NP form close-packed clusters of various sizes. Field of view is 2 μm × 2 μm.

The inherent metabolic stress response of *E. coli* cultures is used to transduce the presence of heavy metal ions in water into metabolites. We then fingerprint the metabolic response with a combination of SERS detection and ML analysis (SERS +ML). *E. coli* cultures were exposed to Cr^6+^ or As^3+^ ions (K_2_Cr_2_O_7_ or NaAsO_2_) in minimal media for 2 h (Fig. 1*A*). Metabolites from the cells were extracted by thermal lysis, and the lysate was deposited on SERS surfaces composed of Au NP clusters for spectral data acquisition (Fig. 1 *B* and *C*). SERS surfaces were fabricated in microfluidic channels with electrodes in a capacitor architecture to achieve reproducible billion-fold signal enhancements (Fig. 1 *E* and *F*) (27). SERS spectra of control samples prepared under the same conditions without Cr^6+^ or As^3+^ in the exposure medium were used to determine the limit of blank (LOB) (33). The full concentration range of samples was collected over the course of several experiments. Each subset of concentrations was collected with a control group included which was not exposed to any metal. To avoid training the algorithm to classify based on background fluctuations, inherent biological variation, or manufacturing variations of SERS surfaces, control samples were measured in biological duplicates and on multiple SERS surfaces (see *Methods* for more details).

The exposure of bacterial cultures to toxic metal ions is expected to result in significant changes in metabolite concentrations. Such metabolic shifts resulting from stress responses often involve differential regulation of nucleotides central to biosynthetic processes within the cell. Metabolic changes in response to antibiotic stress have been reported to be detectable within 30 min of exposure by mass spectrometry (3). Some metabolic stress responses are general, for example, those triggered by the sigma factor regulon, RpoS, which can be regulated by proteins dependent on concentrations of the nucleotide adenosine triphosphate (ATP) (34). ATP accumulates in *E. coli* as part of its stress response to antibiotics (35) and ATP-coupled pumps are associated with As^3+^ transport out of cells in response to toxic exposure (36). Uracil, another nucleotide, is a building block of RNA and thus related to protein translation, and its concentration is closely correlated with oxidative stress responses in bacteria (3, 37). Another nucleotide, adenine, regulates the cell cycle in bacteria, including cell division and DNA repair, and processes modulated in stress conditions (38). To verify that SERS surfaces are sensitive to these and similarly Raman active metabolites associated with bacterial stress response, SERS spectra of 1 mM aqueous solutions of key nucleotides ATP, uracil, and adenine were acquired, and representative spectra are shown in Fig. 1*D*.

### Training Data Acquisition for Fingerprinting Bacterial Stress Response.

SERS spectra were acquired from lysate from *E. coli* cells exposed to heavy metal ion solutions at various concentrations untreated (control). The concentration range investigated with SERS + ML for NaAsO_2_ was 0.65 pg/L to 650 mg/L (13 concentrations) and for K_2_Cr_2_O_7_ was 0.1 ng/L to 10 mg/L (9 concentrations). The corresponding molarities are 5 fM to 5 mM for As^3+^ and 0.68 pM to 68 µM for Cr^6+^. The concentration range was chosen to span the WHO recommended limit for these metals in drinking water, which are 10 µg/L (0.13 µM) and 50 µg/L (0.96 µM) for As^3+^ and Cr^6+^, respectively. SERS spectra acquired from pure solutions of Cr^6+^ (6.8 pM) and As^3+^ (0.5 pM) without *E. coli* cells show that the vibrational peaks observed from lysate samples are due to the cellular metabolites instead of heavy metal ions themselves (*SI Appendix*, Fig. S2).

**Fig. 2.**
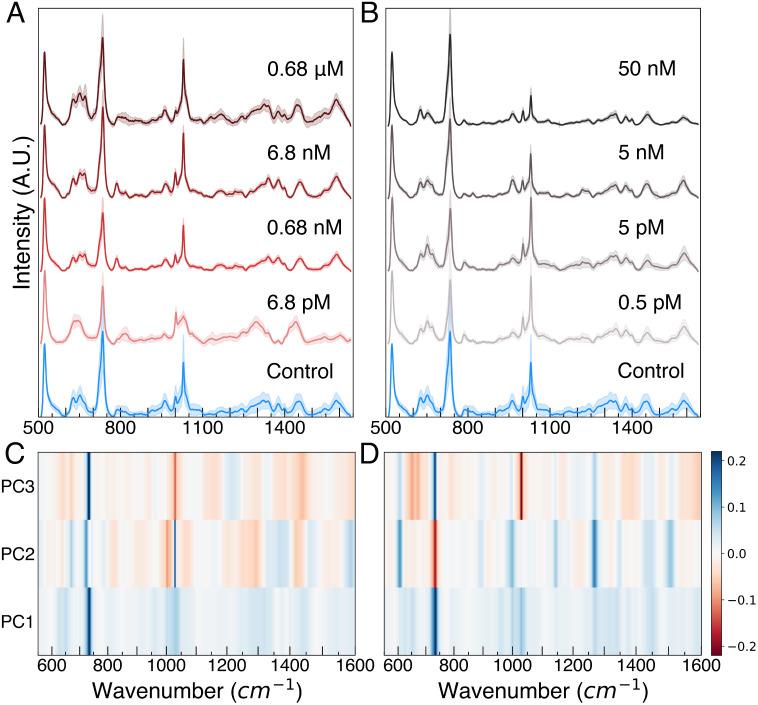
Concentration-dependent averaged SERS spectra (vertically offset with standard deviation shaded above and below each spectrum) acquired from *E. coli* cultured in media with indicated (*A*) K_2_Cr_2_O_7_ and (*B*) NaAsO_2_ concentrations. PC1, 2, and 3 heat map of (*C*) the Cr^6+^ dataset and (*D*) the As^3+^ dataset containing spectra of lysate from control and the full range of metal concentration exposure.

Average SERS spectra of *E. coli* lysate after metal ion exposure show spectral feature differences to the eye (Fig. 2 *A* and *B*). Principal component (PC) analysis (PCA), used for dimensional reduction of SERS spectra, more clearly highlights spectral feature changes associated with different metal exposure conditions. Analysis of the entire spectral range, versus individual peaks, has been reported to improve analysis of SERS data of complex samples (39–41). Before PCA, SERS spectra undergo baseline correction, data smoothing, and normalization (*Methods*). We found that 22 PCA components, shown in *SI Appendix*, Fig. S3, capture 93.3% and 94.8% of variances for Cr^6+^ and As^3+^ concentration data, respectively. The scores are plotted in *SI Appendix*, Fig. S4. In Fig. 2 *C* and *D*, the first three PC loadings, which account for greater than 75% of spectral variance used for sample classification, are shown in a heat map. For example, the heat map of Fig. 2*D* shows the largest loading value of PC1, which accounts for 58% of the variance, between 700 and 750 cm^−1^, which is a band consistent with SERS features associated with DNA methylation (42) associated with the stress response of *E. coli* (43). The stress response to metal toxins involves differential regulation of nucleotides related to biosynthetic processes within the cell. Metabolite vibrational mode assignments are shown in *SI Appendix*, Table S1. The largest loading features in PC1, PC2, and PC3 correlate with energy nucleotides, which are associated with energy metabolism pathways involved in toxic metal stress response in bacteria (44–46), suggesting that changes in nucleotide concentrations in response to metal exposure are consistent with the features upon which the algorithm is classifying the different exposure conditions. Thus, this platform is promising to identify biochemical networks involved in toxin stress response when combined with network models as performed by Yang et al. to identify metabolic mechanisms of antibiotic lethality (47).

### Classifying Lysate Spectral Concentrations by SVM.

We hypothesized that while differences in lysate spectra associated with heavy metal exposure might be difficult to identify by eye, ML algorithms could accurately classify these differences as a function of metal concentration. An unsupervised ML algorithm, t-distributed stochastic neighbor embedding (tSNE), is used for comparing similar data points in lower dimensional space. The tSNE plots show clear differences in the spectral data that correlate with exposure concentration (*SI Appendix*, Fig. S5). These plots represent preliminary validation of our hypothesis that the differences in metabolic responses observed in the cell lysate are evident in spectral data and not a result of algorithm training. These components are used as inputs for training two independent SVM discriminative ML models, one for Cr^6+^ and one for As^3+^, in order to demonstrate the ability to accurately distinguish different heavy metal exposure concentrations as a means to evaluate water safety. The classes in each discriminative model are the concentrations of metal ions: the model for Cr^6+^ has 10 classes (for nine metal concentrations + control) and for As^3+^ there are 14 classes (for 13 metal concentrations + control).

**Fig. 3.**
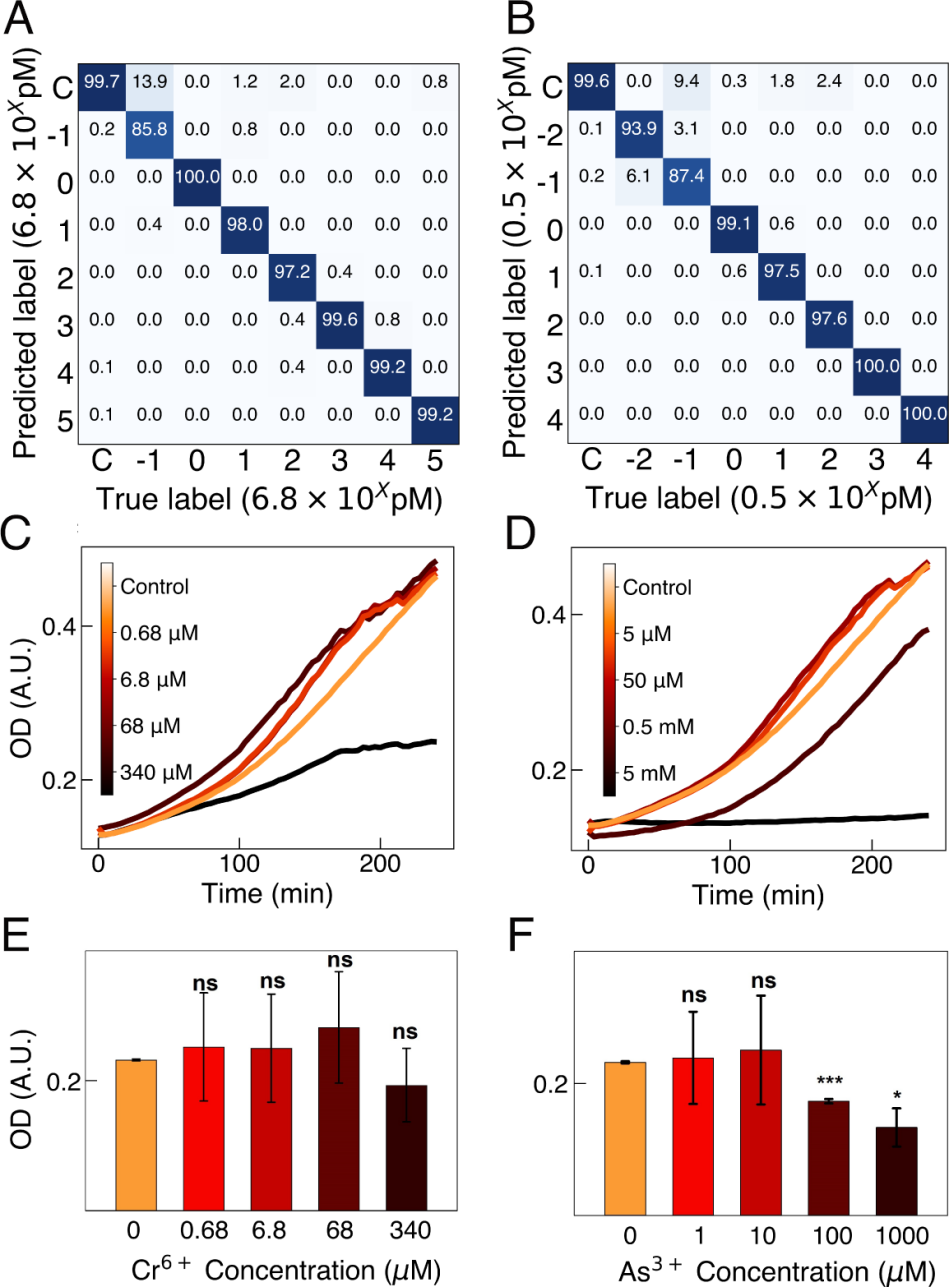
Classifying lysate spectral concentrations. (*A*) SVM confusion matrices showing accuracy of classifying of different concentrations of Cr^6+^ (label scale bar is on a log scale in units of 6.8 pM) and (*B*) As^3+^ (label scale bar is on a log scale in units of 0.5 pM) in the correct concentration class. Growth curve for (*C*) Cr^6+^ and (*D*) As^3+^ at different exposure concentrations. Corresponding OD from the growth curves at 2 h for different concentrations of (*E*) Cr^6+^ and *F* As^3+^, where ns = no significant difference between the experimental groups and control, *P ≤ 0.05, **P ≤ 0.01, and ***P ≤ 0.001. Experiments were done in biological duplicate.

The training datasets are imbalanced since the size of the control class (measured in biological duplicate) dataset (9,600 spectra) is eight times larger than the classes corresponding to a single concentration (1,200 spectra). The synthetic minority over-sampling technique (SMOTE) is a standard method to manage imbalanced data sets by performing data augmentation (*Methods*) (48). SMOTE is performed after dataset division to prevent data leakage. The model is trained with 80% of the spectral data, and the resulting classification accuracy is determined by algorithm predictions on a holdout set (not seen by the SVM model during training) composed of the remaining 20% of the data. The classification accuracy of the holdout set is plotted in the confusion matrices for Cr^6+^ (Fig. 3*A*) and As^3+^ (Fig. 3*B*). The concentration label of Cr^6+^ and As^3+^ datasets is transformed to logarithmic scale. The LOD was determined to be at the value when the prediction accuracy was higher than 98% in distinguishing from the control sample. At concentrations of 6.8 pM for Cr^6+^ and 0.5 pM for As^3+^, there are less than 0.3% false predictions of control rather than the true concentration (Fig. 3 *A* and *B*). Thus, SERS + ML yields a LOD of 6.8 pM for Cr^6+^and 0.5 pM for As^3+^. The SVM classification model was also evaluated by traditional sensor performance metrics of sensitivity, specificity, and accuracy (*SI Appendix*, Table S2). Overall, above the LOD, the sensitivity, specificity, and accuracy are all higher than 97% for both As^3+^ and Cr^6+^. In order to put these metrics in perspective, we compare the analysis from SVM models to analysis of the culture optical density (OD) data (Fig. 3 *C*–*F*) used for assessing cell growth and inhibition by stressors. There is no significant difference in culture OD 2 h after exposure to Cr^6+^ even at concentrations of 340 µM, and there is a significant difference in OD for As^3+^ compared to control only at concentrations greater than 100 µM. At an OD of 0.5, the LOD determined from the SVM model corresponds to approximately 0.6 As^3+^ ions per bacterium in solution and 8.2 Cr^6+^ ions per bacterium in solution. This correlates well with the recommended safe concentration of Cr^6+^ being 10 times higher than As^3+^. Thus, SERS + ML achieves six orders of magnitude lower concentration detection versus methods based on growth inhibition.

### Classification of Type of Heavy Metal Ion Contaminants.

**Fig. 4.**
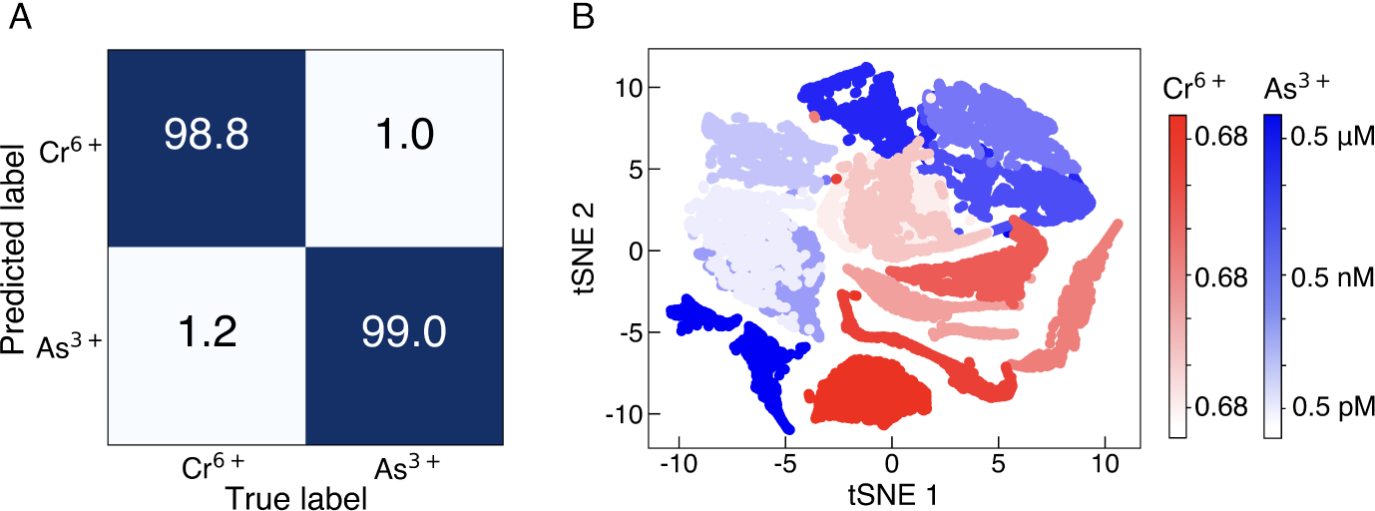
Investigation of different types of heavy metal ion contamination. (*A*) SVM confusion matrix for classification between Cr^6+^ and As^3+^ for concentration range 0.68 pM to 0.68 µM and 0.5 pM to 0.5 µM, respectively. (*B*) tSNE clustering analysis for different concentrations of Cr^6+^ and As^3+^ in red and blue, respectively.

We hypothesized that the metabolic consequences of As^3+^ and Cr^6+^ exposure should be differentiable by SERS + ML of cell lysate due to differences in the mechanism of toxicity of these two metals. An SVM binary classification model was trained on lysate from cells exposed to Cr^6+^ at concentrations in the range of 0.68 pM–0.68 µM and As^3+^ at concentrations 0.5 pM–0.5 µM, at 10-fold concentration increments. These ranges span the LOD achieved with SERS + ML for each of the two metals. The algorithm training process follows an analogous flow (baseline correction, smoothing, normalization, data reduction) as described for the classification of concentration in the prior section (*Methods*). Using this approach, Cr^6+^ and As^3+^ contamination can be distinguished with a high classification accuracy of 98.8% (Fig. 4*A*). The ability to distinguish between different types of heavy metal ions in water is of great importance for determining the pollution source and water treatment process. Analysis of the two metal data sets with tSNE shows that there are clear differences in spectral data even when the data are not labeled during training (Fig. 4*B*).

### CNN Regression for Sensitive Quantification of Heavy Metal Concentrations.

**Fig. 5.**
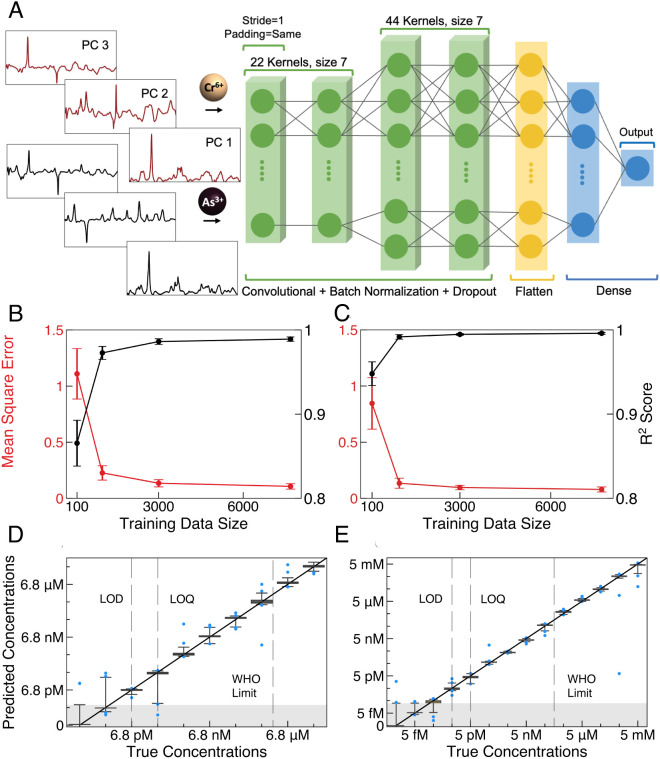
1D CNN regression model for quantitative concentration determination. (*A*) Schematic of process flow in training 1D CNN architectures using 22 PC from Cr^6+^ and As^3+^ concentration data. The 1D CNN model is 4 layers deep. The flatten layer is used to convert the data into a 1D array for inputting it to the fully connected dense layer. The output layer has one node with linear activation function to produce a predicted value. The MSE and R^2^ variance as a function of training class size for (*B*) Cr^6+^, and (*C*) As^3+^. The training data size from each class is 100, 1,000, 3,000, and 7,680. Each training algorithm runs 10 times to generate a mean value and SD for MSE and R^2^. CNN regression boxplots for (*D*) Cr^6+^ and (*E*) As^3+^. Boxes contain 50% of predicted concentration values, and vertical lines indicate the range containing 99% of predicted concentration values. Blue dots show the remaining 1% outliers.

In addition to evaluating how SERS + ML is able to assign a concentration to a particular class (Fig. 3), we also demonstrate that algorithms can predict the actual concentration of heavy metal ions in water. Monitoring concentration changes below Environmental Protection Agency (EPA) regulatory and WHO recommended limits is important for early detection of contaminants entering water supplies before adverse effects occur. CNN was used for regression analysis as it outperforms SVM in terms of throughput and regression error (49). Two independent 1-dimensional (1D) CNN regression models are trained on Cr^6+^ and As^3+^ concentration-dependent cell lysate spectral data. The same 10 and 14 metal concentration classes for Cr^6+^ and As^3+^, respectively, were used as before (Fig. 3). The CNN model architecture (Fig. 5*A*) contains four 1D convolutional layers with inputs of 22 PCA components representing the Cr^6+^ and As^3+^ concentration data. The first convolutional layer has the same padding and a stride of 1 to preserve the spatial dimensions of the input data. Each convolutional layer uses a rectified linear (ReLU) activation function and is followed with batch normalization and dropout with 20% random dropout rate to avoid overfitting (*Methods*). As before, the spectral data are baseline corrected, smoothed, normalized, and dimensionally reduced using PCA before input into the model. The holdout set for validation is composed of 20% of the data, and the remainder is used for training.

First, we use 10-fold cross-validation for hyperparameter tuning and model performance evaluation. The number of epochs (training cycles) in the 1D CNN was determined by monitoring the convergence of the training and validation loss. The loss function is calculated to determine the mean square error (MSE) error between the predicted values and the true values. As one can see in the *SI Appendix*, Fig. S8, the algorithm converges to a loss value of approximately 0.1 at an epoch of 35. In order to utilize SERS + ML for a variety of contaminants in practice, it is important to evaluate required data set size achieving accurate results. A randomly chosen subset of the data composed of 100 spectra per class is first analyzed. The coefficient of determination (R^2^) of linear regression was also calculated as a complementary metric to MSE to evaluate model performance (50). MSE and R^2^ score were calculated as a function of training data size and plotted in Fig. 5 *B* and *C*. As one can see the MSE (R^2^ score) values are high (low) for this smaller dataset and exhibit high fluctuations. The training dataset includes 960 spectra per class per exposure condition, this requires 10 min for acquisition. The control dataset contains 7,680 spectra. As before SMOTE is used for data augmentation for the concentration classes to balance with control data. When the training dataset has 1,000 spectra per class, which contains only 40 generated spectra, the model achieved an MSE value of 0.17 (0.23) for As^3+^ (Cr^6+^) and R^2^ score of 0.98 (0.97) for As^3+^ (Cr^6+^). If further augmentation is performed using SMOTE to produce 7,680 spectra per class to balance with control, the MSE reduces to 0.09 (0.11) for As^3+^ (Cr^6+^) and R^2^ score increases to 0.99 for both As^3+^ and Cr^6+^. Thus, we can achieve robust model performance using SERS spectra, which can be acquired rapidly.

The 1D CNN regression model performance on the balanced data set is plotted in Fig. 5 *D* and *E*. The results are presented as box plots where the data in the boxes contain 50% of the predicted values of the holdout data, vertical lines extend to include up to 99% of predicted values, and the remaining outliers are represented individually by blue dots. The narrow height of the box plots show that SERS + ML provides concentration quantification with high precision. The gray shaded region at the bottom of figures highlights the LOB. The resulting LOD is highlighted with a vertical dashed line and is defined as having less than 0.5% overlap with control data. The values are in agreement with that determined by the SVM model (Fig. 3 *A* and *B*) demonstrating robust performance of SERS + ML regardless of algorithm type. The 1D CNN regression model also allows for determining a limit of quantification (LOQ), highlighted with a vertical dashed line, where the overlap between neighboring concentrations is less than 0.5%. The values of LOQ are 68 pM for Cr^6+^ and 5 pM for As^3+^. The dynamic range spans from the LOQ to 68 µM for Cr^6+^ and LOQ to 5 mM for As^3+^. Chronic exposure at doses of 50 µg/L of arsenic in drinking water is correlated with disease, such as cancer (51). In addition to regulatory limits, the US EPA defines a maximum contaminant level goal in drinking water that is known to have no adverse effects on the health of people. For arsenic, this value is zero. The EPA regulatory limit (10 μg/L for As^3+^ and 100 μg/L for Cr^6+^) is the value that is enforceable and provides a buffer for health safety. There is value, therefore, in detection at concentrations lower than the regulatory limit.

### Determination of Contaminant Levels in Tap Water and Wastewater Samples.

**Fig. 6.**
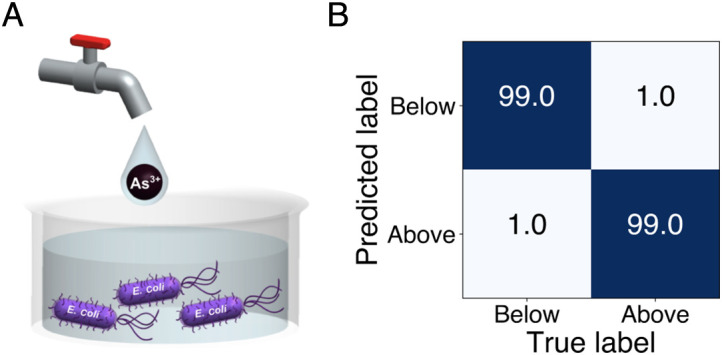
Performance of SERS + ML on unseen tap water samples. (*A*) *E. coli* is cultured in growth media and added to tap water supplemented with As^3+^ salts at concentrations of 1.3 nM, 13 nM, and 1.3 µM for 2 h. (*B*) CNN confusion matrix of binary classification of spectral lysate data exposed to tap water at concentrations above and below WHO standard for drinking water for As^3+^.

Water samples from different sources unseen by the trained algorithm are analyzed to demonstrate that SERS + ML is generalizable. Drinking water, water used in agriculture, and wastewater will contain different types of impurities, which may perturb the stress response of *E. coli*. It is not feasible to fully train a new model for every different water sample. Transfer learning is an effective method to analyze similar systems with small datasets while still achieving high prediction accuracy. During transfer learning, the weights and bias of the first and second convolutional layers are adjusted and other layers are fixed. In practice, this method could be applied by spiking contaminants in water samples for fine-tuning the model for the water sample of interest. In order to demonstrate this principle, a 1D CNN model was pretrained with spectra from deionized (DI) water samples spiked with As^3+^ at 0.05, 0.5, and 5 nM (below WHO recommended level) and 5, 50, and 500 µM (above WHO recommended level). Then, unseen tap water samples are spiked with As^3+^ at concentrations of 1.3 nM, 13 nM, and 1.3 µM. A binary model is assembled to predict if tap samples contain As^3+^ above or below WHO recommended levels. The number of spectra per class needed to fine-tune the model is 80, which takes only 2 min of acquisition time for the entire training dataset. The results are shown in Fig. 6*B* where the model was able to categorize tap water samples as above or below regulatory limits with 99% accuracy. It is worth noting that the different As^3+^ concentrations in the tap water samples is not the same as in the DI water samples. This is important to determining accuracy of evaluating unknown samples.

**Fig. 7.**
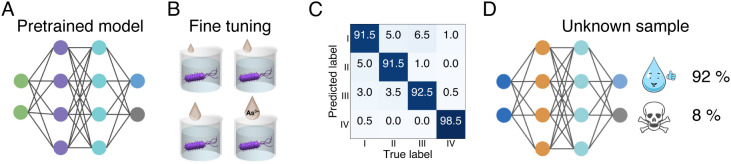
Performance of SERS + ML on unseen wastewater samples. (*A*) The model is pretrained on DI water (*B*) is fine-tuned with waste water samples spiked with (I) 1.3 nM, (II) 13 nM, (III) 1.3 µM, and (IV) 13 µM As^3+^. (*C*) The accuracy of differentiating the different As^3+^ concentrations in spiked wastewater samples after pretraining. (*D*) The fine-tuned model is able to determine that the concentration of As^3+^ in the original wastewater sample is below the WHO recommended level with 92% accuracy.

In order to analyze more complex samples, As^3+^ was also spiked in secondary treated wastewater from a local wastewater treatment plant. These samples are more complex as they contain heavy metal contaminants in the background. *SI Appendix*, Table S3 shows the primary pollutant analysis summary from the sanitation district where the As concentration in the background is approximately 19.4 nM. The process of determining if the concentration in the unspiked sample is above or below WHO level for As is shown in Fig. 7. Wastewater samples are spiked with concentrations of 1.3 nM, 13 nM, 1.3 µM, and 13 µM. Again spanning above and below WHO recommended levels, 130 nM, for model fine-tuning of the above pretrained DI model used for tap water. Fig. 7 shows classification accuracy of differentiating the different classes used for training. When applying the model to the unspiked sample, the model predicts that the As^3+^ concentration is below WHO level with 92% accuracy. The total data acquisition time is 8 min; thus, acquiring samples in the field to fine-tune a model in a short amount of time produces high accuracy.

## Discussion

The *E. coli* whole-cell sensors are shown to transduce metal ions into chemical signals using the inherent metabolic stress response. Robust and sensitive SERS surfaces with high enhancement factors (21, 27, 30) are able to gather large, reproducible datasets needed for ML analysis. The dataset size per class for training and validation is composed of 1,200 spectra, which requires 10 min when using the SERS surfaces developed by the authors. Thus, we can achieve robust model performance using SERS spectra which can be acquired rapidly. Changes in the metabolite profile in *E. coli* cell lysate associated with a stress response to heavy metal toxins in water are observable in SERS spectra even when using unsupervised feature extraction methods such as tSNE, which computes similarity of data in lower dimensional space. There are clear differences in the spectral response across the entire range of concentrations to which cells were exposed (*SI Appendix*, Fig. S5). These plots represent validation of our hypothesis that the differences in metabolic responses observed in the cell lysate are evident in spectral data and not a result of algorithm training.

When using SVM, a supervised algorithm, for data analysis, the resulting changes in metabolite concentrations in *E. coli* cell lysate are observable in SERS spectra and differentiable across exposure concentrations with a dynamic range of 10^5^ (Fig. 3). The spectral changes are distinct from control samples (unexposed) down to concentrations at which the number of As^3+^ in solution per cell is approximately 1. For Cr^6+^ exposure, this number is approximately 10 ions per cell. These values correlate well with the fact that the EPA regulatory limit of Cr^6+^ is ten times higher than As^3+^. Overall, the LOD of SERS + ML is 100,000 lower than the WHO recommended and US EPA regulatory levels (Fig. 3). Detection well below regulatory limits is beneficial because the EPA maximum contaminant level goal for As^3+^ is zero. Consequently, this platform is promising for monitoring changes in water quality below regulatory limits to provide early warning of water contamination and accurate longitudinal tracking of contaminant concentrations. The metabolite changes detected by this system can also distinguish between Cr^6+^- and As^3+^-induced responses in water with a classification accuracy of 99% (Fig. 4). Identifying the type of metal contamination is critical to locating the source and determining necessary treatment (52). When using 1D CNN regression algorithms, the LOQ is 68 pM for Cr^6+^ and 5 pM for As^3+^ with a dynamic range of 6 orders of magnitude (Fig. 5). The 1D CNN regression model yields the same LOD as SVM (Fig. 3 *A* and *B*) demonstrating robust performance of SERS + ML regardless of algorithm type.

Monitoring the quality of tap water and water discharged from water treatment facilities will require analysis of samples with a distribution of impurities, which may perturb the stress response of *E. coli*. It is not feasible to fully train a new model for every type of water sample in the field. Transfer learning is shown to be an effective method to analyze similar systems with smaller training datasets while still achieving high prediction accuracy. By obtaining water samples and spiking with known concentrations of contaminants, a new model can be quickly fine-tuned with a smaller data set. Transfer learning using data obtained in several seconds is sufficient to determine if drinking water or wastewater is unsafe (Fig. 6), i.e., above or below WHO recommended limits with greater than 96% accuracy. For more complex samples, secondary treated wastewater, the fine-tuned models can determine if the unspiked waste water sample is above or below recommended safety limits with 92% accuracy. While here we demonstrated that transfer learning is an effective way to evaluate one type of metal contaminant in an ‘unknown’ samples with multiple background contaminants, we envision an assay approach could be used to examine water samples for the presence of other toxins. Overall, we demonstrate that trained algorithms are rapidly generalizable across different water samples. The whole-cell SERS + ML platform is promising for application to other water sources, such as recycled water, and to other metals of concern such as lead, mercury, and cadmium.

## Materials and Methods

### Sensor Fabrication.

SERS surfaces are fabricated in microfluidic channels with a capacitor architecture to apply an AC potential across electrodes (Fig. 1) to induce electrohydrodynamic (EHD) flow. Fabrication is performed silicon substrates (NOVA Electronic Materials, P-type, boron doped <100> with resistivity of 0.001 to 0.005 Ω cm) with dimensions of 15 mm × 15 mm that are spin coated with poly(styrene-b-methyl methacrylate) (PS-b-PMMA, Mn S-b-MMA 170000-b-145000 g mol^−1^) thin films of approximate thickness of 25 nm; Si substrates serve as the working electrode. Indium tin oxide (ITO)-coated glass slides (Delta Technologies) serve as the counter electrode. EHD, which results as Au NPs attach to the working electrode and locally perturb the surface potential, is used as an external driving force for cross-linking reactions between 40 nm lipoic acid-functionalized Au NPs (Nanocomposix, 0.13 nM) to form the anhydride linking group, which define nanogap spacings. Chemical cross-linking reactions between NP leads to Au NP clusters with reproducible SERS signal over a large area (28).

Silicon substrates were cleaned by 20% v/v hydrofluoric acid (HF, Fisher Scientific, 48%) / DI water (Milli-Q Millipore System, 18.2 MΩ cm^−1^) for 5 min to remove the native oxide layer and then immersed in DI water to regrow a thin oxide layer. *The potential of HF to cause severe injury mandates extreme caution during usage*. Random copolymer poly(styrene-co-methyl-methacrylate)-ɑ-hydroxyl-ω-Tempo moiety (PS-r-PMMA, Polymer Source, Mn = 7,400, Mw = 11,800, Mw /Mn = 1.60, 59.6 mol% polystyrene content) random copolymer dissolved in toluene (Fisher Scientific), 1 wt%, was spin-coated at 3,000 rpm for 45 s on silicon substrates. PS-r-PMMA films were annealed under vacuum at 170 °C for 48 h followed by a rinse with toluene to leave a brush layer. PS-b-PMMA is spin coated at 5,000 rpm for 45 s and then annealed for 72 h at 170 °C. In order to selectively functionalize PMMA domains on PS-b-PMMA diblock copolymer films with amine functional groups for cross-linking with Au NPs, PS-b-PMMA/Si were immersed in dimethyl sulfoxide (DMSO, Sigma-Aldrich) for 5 min and then 5 % vol ethylenediamine (ED, Sigma-Aldrich) in DMSO for another 5 min. ITO counter electrodes were cleaned using ethanol (Sigma-Aldrich), isopropyl alcohol (IPA), and DI water and then dried using N_2_ before attaching a platinum wire and silver paste (Epoxy Technology) to make electrical contact.

A microfluidic cell was formed between electrodes using a 90-µm spacer layer composed of 3M 9816L. A solution of 2 µL N-hydroxysulfosuccinimide (s-NHS, Sigma-Aldrich), 20 mM, and 2 µL 1-ethyl-3-(3-dimethyl aminopropyl) carbodiimide (EDC, Sigma-Aldrich), 8 mM, in a 2-(Nmorpholino) ethane sulfonic acid buffer (MES, Sigma-Aldrich, 0.1 M, pH = 4.7) was added to a 0.25 mL solution of 2.6 nM lipoic acid-functionalized Au NP solution. Then, 20 µL of the solution containing Au NP, s-NHS, and EDC is added to the microfluidic cell. An AC electrical stimuli with a potential of 5 V_p_ and frequency of 100 Hz is applied for 2 min to deposit a seed layer to induce EHD flow. The second deposition step was conducted at a potential of 5 V_p_ and frequency of 1,000 Hz for 2 min to grow Au NP clusters. After deposition, the electrode cell was dismantled and the sensor surface was thoroughly rinsed with DI water and IPA (Sigma-Aldrich) and then dried with N_2_. *SI Appendix*, Fig. S9 shows reproducible intensity across the SERS surface and *SI Appendix*, Fig. S10 compares to intensity from a benzenethiol monolayer obtained from samples fabricated using EHD and drop casting, where the latter has lower signal and highly variable intensity.

### Media, Heavy Metal, and Carbon Source Supplement.

M63 media (VWR Life Science) solution was made by first diluting 1 liter of presterilized M63 5× (BioWORLD, GeneLinx International Inc.) stock solution using autoclaved Millipore water. Filter-sterilized magnesium sulfate anhydrous (MgSO_4_, Fisher Scientific) water solution, of volume 1 mL and molarity of 1 M, was added to the diluted media solution following standard protocol. Sodium arsenate stock solution (RICCA Chemical Company, 100 mM) was first filter-sterilized and then diluted with sterilized DI water to reach concentrations of 0.1 mM and 0.1 µM and stored under 4 °C. Potassium dichromate (Fisher Scientific) solution was made by first dissolving sodium dichromate crystal into sterilized DI water to reach concentrations of 17 mM, and then, the solution was filter-sterilized and diluted with sterilized DI water again to reach concentrations of 0.34 mM and 0.34 µM and stored at 4 °C. Prior to exposure to bacterial cultures, working solutions were placed at room temperature for 30 min to equilibrate to ambient temperature and then titrated to the culture to target exposure concentration. Anhydrous dextrose (glucose, Fisher Scientific), 1 g, was dissolved in 10 mL DI water and filter-sterilized to form 10% (w/v) glucose stock solution, which was added into the media solution later to provide energy source for bacteria.

### Growth and Subculture Condition.

A sterilized wooden applicator was used to streak *E. coli* K12 strain MG1655 (Yale Stock Center via the Goulian Lab) frozen stock onto an lysogeny broth (LB, IBI scientific) agar plate. The plate was then placed into an incubator and incubated stationarily for 18 h. A single colony was picked from the plate after incubation and used to inoculate 5 mL sterile LB solution in a test tube. The inoculated culture tube was then placed in the shaking incubator (I series 24R, New Brunswick) set at 37 °C and speed of 250 rpm for 18 h. After incubation, the final OD was approximately 1.5 as measured with a colorimeter (WPA CO7500 colorimeter, Biochrom Ltd.). From the shaking culture, 3 mL was transferred to a 50 mL conical centrifuge tube and centrifuged at the speed of 5,000 rpm for 5 min (Sorvall Legend X1R centrifuge, Fisher Scientific). Then, the supernatant was disposed and the pellets were resuspended in 1 mL of 1× phosphate-buffered saline (PBS, Fisher Scientific, 10× solution) solution. The pellet-PBS mixture was transferred to 1 mL centrifuge tubes, centrifuged at 5,000 × g for 5 min (accuSpin Micro 17, Fisher Scientific), and the supernatant was disposed. The washing step was repeated. After, the pellet was resuspended in 1 mL M63 defined media, resulting in a milky M63-pellet mixture with very high OD. M63 media supplemented with 1% (w/v) glucose was pipetted into sterilized test tubes and the pellet-M63 mixture was titrated into the test tubes to reach the final OD of 0.5. The total volume of liquid in each test tube was 5 mL. Three tubes, having a 15 mL culture, were prepared for a single colony. These tubes were then moved to the shaking incubator for subculturing with the shaking speed set at 250 rpm and temperature at 37 °C for 6 h. Then, the 15 mL subculture was transferred to 50 mL centrifuge tubes, centrifuged twice at a speed of 5,000 rpm for 5 min, and washed with 1 mL of PBS twice. The subculture was resuspended in 1 mL M63 defined media before being exposed to heavy metals.

### Bacterial Exposure to Heavy Metal and Growth Curve Measurement.

*E. coli* (K12 MG1655 strain) is cultured in defined media M63 to achieve an OD of 0.5 and supplemented with 1% (w/v) glucose to mitigate conflating stress from heavy metal stress ions with nutrition limitation. The subcultures prepared as described in the prior section were washed with 1 mL PBS twice and resuspended in M63 defined media. M63 media supplemented with 1% glucose (w/v) was pipetted into wells of white-opaque 96-well microplates. Different concentrations of heavy metal (NaAsO_2_ or K_2_Cr_2_O_7_) were added to the wells. Specifically, 0, 1, 10, 100, and 1,000 µM of NaAsO_2_ and 0, 0.34, 3.4, 34, and 170 µM of K_2_Cr_2_O_7_ were exposed to cultures for 2 h. The resuspended culture was pipetted into the wells to make the OD of the culture 0.5. Each condition was done in biological duplicates. After pipetting, the microplate was placed in the SkanIt Microplate Reader (Thermo Scientific) at 37 °C and shaken at a speed of 300 rpm and high force. The OD of the culture in each well was measured every 5 min for 6 h to generate growth curves.

Preparation of cultures exposed to tap water and wastewater from Orange County Sanitation District (OCSD) involves similar steps as those exposed to DI water spiked with As^3+^, except after washing with PBS, the subculture was resuspended in tap water or wastewater supplemented with 1% (w/v) glucose at an OD of 0.5, and the heavy metal salts were dissolved in tap water or wastewater instead of the defined media. The secondary treated wastewater was treated by primary sedimentation followed by an activated sludge process with nitrification and denitrification at OCSD. Before spiking with As^3+^, the secondary treated wastewater was filtered with 0.45 µm MCE Membrane (MF-Millipore).

### Lysate Sample Preparation.

Thermal lysis was chosen for our sample preparation process due to its convenience, minimal equipment requirements, speed, and extensive prevalence in microfluidic devices as a method for bacterial membrane disruption (53–59). While thermal treatments can influence the metabolite profile of a sample (60), every method of cell disruption has some effect on cellular contents associated with it (53, 54), and in this study, we only aim to show that machine learning analysis of whole-cell sensors accurately distinguishes between identically prepared samples.

After exposure to metal solutions, the bacterial cultures were washed, as described in the growth and subculturing methods section, to remove residual metals from the pellet and avoid their mixing with metabolites released during lysing. The pellet was then resuspended in 100 µL Millipore water and heated to a temperature of 97 °C for 30 min to lyse the cells. The lysed culture solution was centrifuged at 12,000 × g for 10 min. Then, 100 µL supernatant in each tube was evenly divided into four parts by pipetting into four different 1 mL sterile centrifuge tubes, 25 μL each transfer. These supernatant samples were placed in the −20 °C freezer to store for further analysis.

### Data Acquisition.

Spectral data of lysate samples are acquired by placing a droplet with a volume of 25 µL of lysate from *E. coli* cells untreated (control) or exposed to heavy metal ion solutions at various concentrations on SERS surfaces. The measured concentration range for NaAsO_2_ was 0.65 pg/L to 650 mg/L (13 concentrations) and for K_2_Cr_2_O_7_ was 0.1 ng/L to 10 mg/L (nine concentrations) spaced by one order of magnitude as shown in Table 1. The corresponding concentrations in molarity of As^3+^ and Cr^6+^ are shown in Table 1.

**Table 1.** Cr^6+^ (10 classes) and As^3+^ (14 classes) for machine learning models

	1	2	3	4	5	6	7	8	9	10	11	12	13	14
Cr^6+^	C	0.68 pM*	6.8 pM*	68 pM*	680 pM^†^	6.8 nM^†^	68 nM^†^	680 nM^‡^	6.8 µM^‡^	68 µM^‡^	X	X	X	X
As^3+^	C	5 fM^§^	50 fM^§^	500 fM^¶^	5 pM^¶^	50 pM^¶^	500 pM^¶^	5 nM^¶^	50 nM^¶^	500 nM^#^	5 µM^||^	50 µM^||^	500 µM^||^	5 mM^||^

C is the control class.  Superscripts indicate SERS data acquired on the same SERS surface.

For each exposure concentration, a dataset of 1,200 SERS spectra is acquired using a Renishaw InVia™ micro Raman system with an integration time of 0.5 s, 146 µW laser power at 785 nm excitation wavelength, and a 60× water immersion lens with 1.2 NA (beam diameter of 292 nm). Raman maps were acquired in an array of 20 × 20 with 3 µm steps between measurement points, resulting in 400 spectra per map. Three maps were acquired over different regions of the sample surface resulting in a total of 1,200 spectra per concentration for each metal ion defining a class for initial training of machine learning algorithms (61). The dataset acquisition takes 10 min, and the droplet does not evaporate during this period of time. In order to ensure that the algorithm is not being trained to detect batch-to-batch variations of SERS surfaces, concentration classes between two and six, including control samples, were acquired on different regions of the same SERS surface (droplets exposed to isolated regions), indicated by superscripts in Table 1. Furthermore, the control group, prepared under the same conditions in the absence of Cr^6+^ or As^3+^ exposure, was measured from lysate samples prepared in biological duplicates on different days, from the eight different SERS surfaces, also fabricated on different days, used for the other metal concentrations exposure conditions to train algorithms to not identify differences based on normal variability of experimental conditions such as culture growth, device fabrication, and processing steps.

### Preprocessing of SERS Spectra Data.

For data preprocessing, asymmetric least square correction is utilized for baseline correction, and a Savitzky–Golay filter is used for data smoothing. In order to normalize the data, the vibrational band of silicon at 520 cm^−1^ is used as an internal standard and set to 1. The diblock copolymer layer, between Si and NP clusters, is 25 nm thick, and thus, Si surfaces are not affected by the signal enhancement of Au NP clusters. The metal ion concentration unit was labeled with a log scale since concentrations investigated span several orders of magnitude. PCA was performed for dimensional reduction. We determined that 22 PCA components captured 93.3% and 94.9% of variances for Cr^6+^ and As^3+^ concentration data, respectively. tSNE was also performed to visualize the concentration data in lower dimensional space and show that there are spectral differences in the data observed without labeling data for algorithms.

### SVM Classification Model.

Two independent SVM discriminative models are trained on Cr^6+^ and As^3+^ exposed lysate spectra data for the classes shown in Table 1. The training datasets are imbalanced since the size of the control class dataset (9,600 spectra) is eight times larger than the classes corresponding to a single concentration (1,200 spectra). The SMOTE is used to oversample skewed classes in the dataset and achieve a balanced dataset. SMOTE works by selecting a random example from the minority class, and then, k of the nearest neighbors for that example is found. A randomly selected neighbor is chosen and a synthetic example is created at a randomly selected point between the two examples in feature space. SMOTE can alleviate overfitting by increasing stability with respect to random fluctuations and thereby increase the generalization capability of the classifier (35). SMOTE is performed after data split within each cross-validation fold to prevent data leakage.

The SVM models are trained using 22 PCA components. A holdout set is composed of 20% of the data that is used for final validation and not seen at all during training. The model is trained with the remaining 80% of the spectral data labeled with their appropriate class to define a hyperplane separating data into the correct classes. SVM models are trained with Scikit-learn using default parameters, with radial basis function kernel, Margin parameter (C) = 1, and γ = scale. In order to evaluate SVM model performance, sampling cross-validation is performed using 10-fold stratified sampling on the training dataset for the initial evaluation of model performance. Here, each fold is shuffled and used as validation data to estimate prediction accuracy. The cross-validation results are in the *SI Appendix*, Figs. S6 and S7. The final model is trained with 80% training data and tested with 20% holdout set.

### Statistical Analysis.

The statistical significance between the OD when exposed for 2 h to different heavy metal concentrations (Fig. 3 *E* and *F*) was calculated using two-tailed Student’s *t* test. All growth experiments were done with biological duplicates (n = 2) in 96-well plates. The OD after 2 h of exposure was calculated as the average of three replicate wells, and the error bars represent the standard deviation of the OD of the three wells. The degrees of freedom for all statistical calculations in the two plots are 2. The *t* values and *P* values are shown in Table 2.

**Table 2.** *Accuracy of SERS + ML measurement of metal concentration: t* values and *P* values of final OD after 2-h exposure to heavy metals

[As^3+^] (μM)	0	0.68	6.8	68	340
*t* value	N/A	0.1677	0.3912	30.5232	5.8938
*P* value	N/A	0.8822	0.7334	0.0011	0.0276
[Cr^6+^] (μM)	0	1	10	100	1,000
*t* value	N/A	0.4158	0.3767	1.0135	1.1834
*P* value	N/A	0.7179	0.7426	0.4175	0.3583

### CNN Regression Model.

The 1D CNN model architecture utilizes Keras framework with Tensorflow backend. Twenty-two PCA components are used as input for both Cr^6+^ (0.68 pM to 68 µM) and As^3+^ (5 fM to 5 mM) exposed lysate spectra datasets, respectively. The first convolutional layer is the data input layer, which has 22 kernels with sizes 7 and 1 stride to preserve the spatial size with the same padding. The second convolutional layer also has 22 kernels with size 7. The third and fourth convolutional layers are identical, with 44 kernels with size 7. Each convolutional layer is followed by a batch normalization layer and a dropout layer with 20% random dropout rate. Batch normalization mitigates changes in the distribution of network activations due to the change in network parameters during training. Dropout layers are used to prevent overfitting. Followed by convolutional layers, a flatten layer is added to reshape the 2D extracted feature into a 1D vector followed by a dropout layer. Fully connected layers with 22 nodes with an L2 norm regularization (0.001) and ReLU activation function are applied to process the 1D vector. Finally, using the linear function, the weighted sum of the flatten layer is condensed into a one-unit neuron containing the prediction result between zero and nine (Cr^6+^) or 13 (As^3+^), where the continuous score supplies predicted concentrations.

Hyperparameters of the 1D CNN regression model including number of hidden layers and units, activation function, dropout rate, batch size, kernel size, and number of epochs are optimized by monitoring training and validation loss during 10-fold cross-validation. To be specific, EarlyStopping was used by monitoring the increase in validation loss to determine the number of epochs. Early termination was determined when the validation loss was increasing for 10 consecutive epochs, indicating that the 1D CNN had reached maximum convergence. During 10-fold cross-validation, they all reach the convergence at approximately 35 epochs, which was thus chosen for the final model. During 10-fold cross-validation, the loss function is calculated to determine the average of the squared differences between the predicted and true values. The overlaid learning curve from 10-fold cross-validation shows no obvious gap between training loss and validation loss, which shows the absence of overfitting (*SI Appendix*, Fig. S8).

Due to the large size of control dataset acquired to capture variability of experimental conditions, including biological culture conditions and device fabrication, the data classes are imbalanced. Again SMOTE is used to balance the training dataset, and here, the training dataset size is varied to contain 100, 1,000, 3,000, and 7,680 randomly selected spectra from each class to determine the size of needed training data for accurate predictions. As before, 20% of the spectral data is set aside as a holdout set, i.e., not used in training. The performance of the 1D CNN regression model is evaluated by calculating MSE and coefficient of determination (R^2^) scores for four different dataset sizes. The R^2^ metric is the ratio of explained sum of squares and the total sum of squares and is sensitive in the order of predicted and actual targets. MSE and R^2^ score mean values and SD are calculated by running the calculations 10 times.

The final CNN model is trained with tuned hyperparameters on 80% of the spectral data (training set), and the model performance is evaluated on the remaining 20% of the spectral data (hold out set), with batch size 44, number of epoch 35, and Adam for gradient descent optimization. The holdout set in the classes is unbalanced where the control class has 1,920 spectra and other classes have 240 spectra. We thus use random downsampling of the control to include 240 spectra to balance the data and represent those in the box plot on Fig. 5 *D* and *E*.

### Transfer Learning.

The transferred CNN is built by Tensorflow 1.8 in Python 3.6. The 1D CNN binary classification model is pretrained to identify the heavy metal concentration in DI water. The classes contain spectra from DI water samples spiked with concentrations of As^3+^ of 0.05 nM, 0.5 nM, and 5 nM (below WHO recommended level) and 5 µM, 50 µM, and 500 µM (above WHO recommended level). The pretrained model is then transferred to identify if the As^3+^ concentration in tap water samples is above or below WHO recommended level. The concentrations tested are 1.3 nM, 13 nM (below), and 1.3 µM (above). For wastewater, the four classes tested contain 1.3 nM, 13 nM (below), 1.3 µM, and 13 µM (above) concentration of As^3+^. The fully connected layer and output layer of the pretrained model are replaced with an output layer which has 1 node with sigmoid activation function. The weights of the third and fourth convolutional layers are frozen throughout fine-tuning, and the weights of the first and second layers are set to be trainable. Before fine-tuning, the model is compiled with binary cross-entropy as loss function, accuracy as metric, and Adam optimizer with a 0.001 learning rate is used. Eighty examples from each class from the new water type are used to fine-tune the compiled transferred model. The performance of the transferred model is tested by 1040 tap water samples. Machine learning algorithm code is available online (62).

## Supplementary Material

Appendix 01 (PDF)

## Data Availability

SERS spectra data present in this manuscript were in txt format. Machine learning algorithms used in the study, PCA, SVM, tSNE, 1D-CNN, and transfer learning, were done using Python in Jupyter Notebook. The full data (https://doi.org/10.5281/zenodo.7109184) and code (https://github.com/hwei77/HeavyMetalML) are available on Zenodo and GitHub, respectively.
