# Supplementary material for: Decoding the metabolic response of Escherichia coli for sensing trace heavy metals in water
Source: Proc Natl Acad Sci U S A. 2023 Feb 6;120(7):e2210061120. doi: 10.1073/pnas.2210061120 (PMC9963153; doi:10.1073/pnas.2210061120)
Supplement: Supplementary file 1 — Appendix 01 (PDF) [file pnas.2210061120.sapp.pdf]

PNAS

Supplementary Information for
Decoding the metabolic response of Escherichia coli for sensing trace heavy metals in water

Hong Wei,[1] Yixin Huang,[2] Peter J. Santiago,[1] Khachik E. Labachyan,[3] Sasha Ronaghi,[4] Martin Paul Banda Magana,[5] Yen H. Huang,[7] Sunny Jiang,[7,8] Allon I. Hochbaum,[1,2,5,6,*] Regina Ragan[1,2,*]

[1] Department of Materials Science and Engineering, University of California, Irvine, Irvine, CA 92697-2585
[2] Department of Chemical and Biomolecular Engineering, University of California, Irvine, Irvine, CA 92697-2580
[3] Department of Pharmaceutical Sciences, University of California, Irvine, Irvine, CA 92697-3958
[4] Sage Hill School, Newport Coast, CA 92657 *current position: Computer Science, Stanford University, Palo Alto, CA 94305
[5] Department of Molecular Biology and Biochemistry, University of California, Irvine, Irvine, CA 92697-2525
[6] Department of Chemistry, University of California, Irvine, Irvine, CA 92697-2025
[7] Department of Civil and Environmental Engineering, University of California, Irvine, Irvine, CA 92697-2175
[8] Department of Ecology and Evolutionary Biology, University of California, Irvine, Irvine, CA 92697-2525

*Regina Ragan
rragan@uci.edu
*co-corresponding: Allon I. Hochbaum
hochbaum@uci.edu

This PDF file includes:

Figures S1 to S10
Tables S1 to S3
SI References

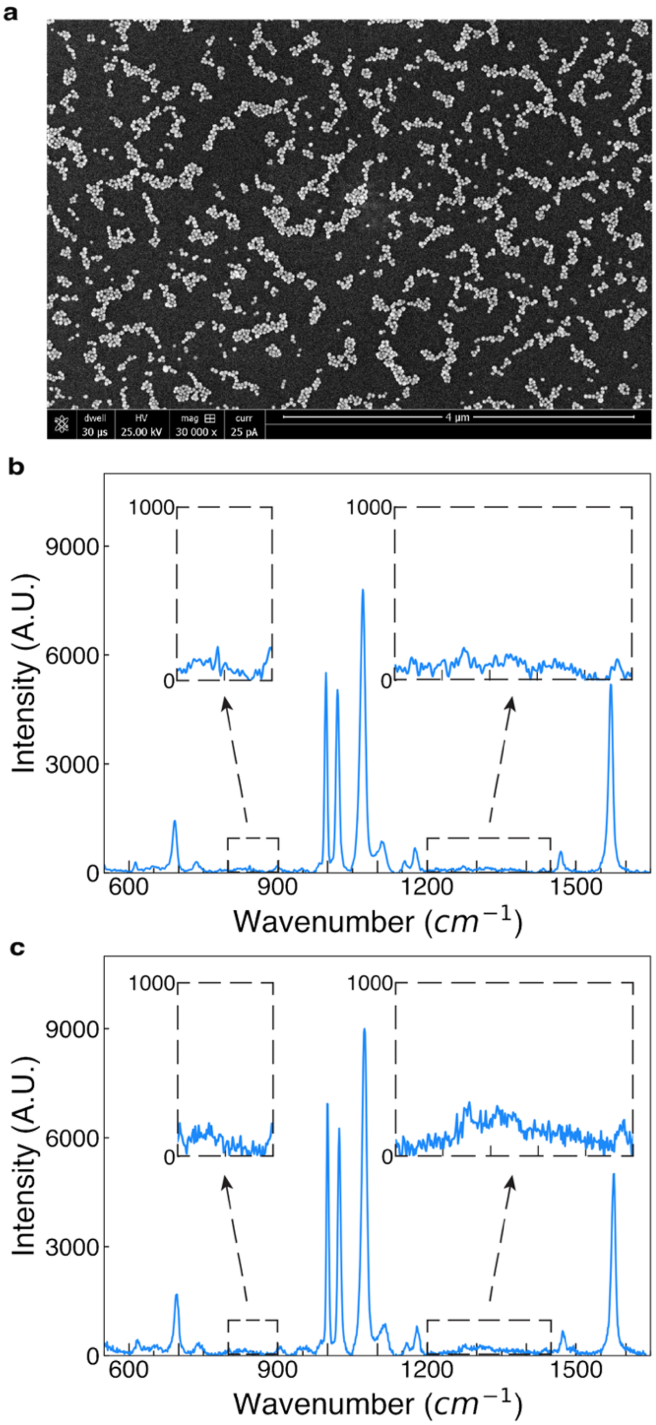

Fig. S1. Comparison of BWTek i-Raman Plus (Model: BWS465-785S) portable spectrometer and Renishaw InVia^{TM} confocal Raman microscope: **a.** Scanning electron microscopy image of the SERS sensor surface with field of view of approximately 30 µm^2, comparable to beam diameter of portable spectrometer. The scale bar is 4 µm. Representative, background subtracted SERS spectra of benzenethiol, a standard Raman reporter molecule, self-assembled monolayer on the same sensor surface acquired with **b.** i-Raman and **c.** Renishaw systems. The regions highlighted with dashed boxes and expanded as insets, 800 - 900 cm^{-1} and 1200 - 1450 cm^{-1}, are reported in prior studies to lack modes with observable SERS signal[1,2] and thus represent background noise used for conventional signal to noise ratio calculations**.**

A self-assembled monolayer of benzenethiol was formed by soaking sensor surfaces in 1 mM benzenethiol in ethanol overnight. We used the intensity of two peaks in spectra from each system, the C-C-C bending + C−S stretching mode (1075 cm^{-1}) and the C-H ring bending mode (1180 cm^{-1}). We chose the latter, as this mode is not always observed in SERS data;[1] the ability to resolve this mode is attributed to the large enhancements from sensor surfaces used here. The root mean square (rms) value of noise was estimated from a baseline region of the spectrum, highlighted with dashed boxes in Fig. S1.b.c., where SERS signal is not observed from benzenethiol,[1,2] using the following equation,[3]

$$rms\ noise = \sqrt{\sum_i (R_i - R_{av})^2/n} \qquad (1)$$

where R_i is the intensity at each pixel in the spectral region, n is the number of pixels, and R_{av} is the corresponding average intensity value. The intensity (I) at 1075 cm^{-1} and 1180 cm^{-1} is used in equation (2):

$$SNR = I/rms\ noise \qquad (2)$$

The SNR using the peak intensity at 1075 cm^{-1} is 181.8 ± 24.9 and 191.0 ± 17.5 and using the peak intensity at 1180 cm^{-1} is 15.1 ± 2.1 and 17.0 ± 1.5 for the Renishaw and iRaman systems respectively.

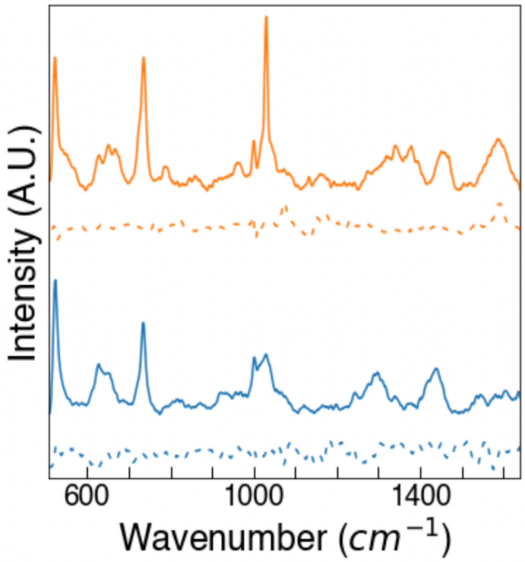

Fig. S2. Surface enhanced Raman scattering spectra from Cr^{6+} (dotted blue) and As^{3+} (dotted orange) salts dissolved in deionized water at concentrations of 6.8 pM and 0.5 pM, respectively. Spectra from bacteria cultures exposed to metals at the same concentration in deionized water are plotted above for Cr^{6+} (blue) and As^{3+} (orange). All spectra are background subtracted.

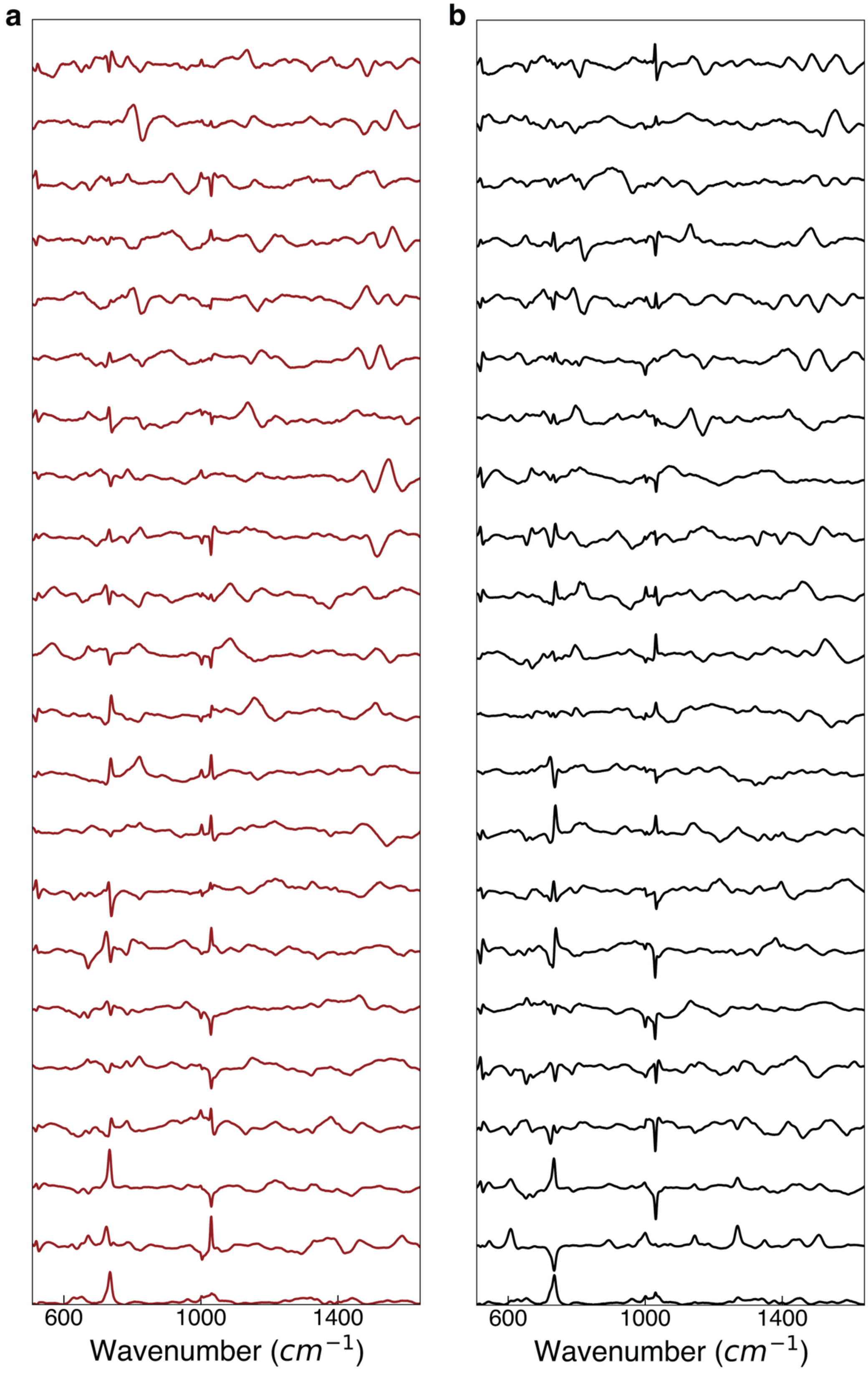

Fig. S3. PC loadings from **a.** Cr^{6+} and **b.** As^{3+}; bottom to top is PC1 to PC 22.

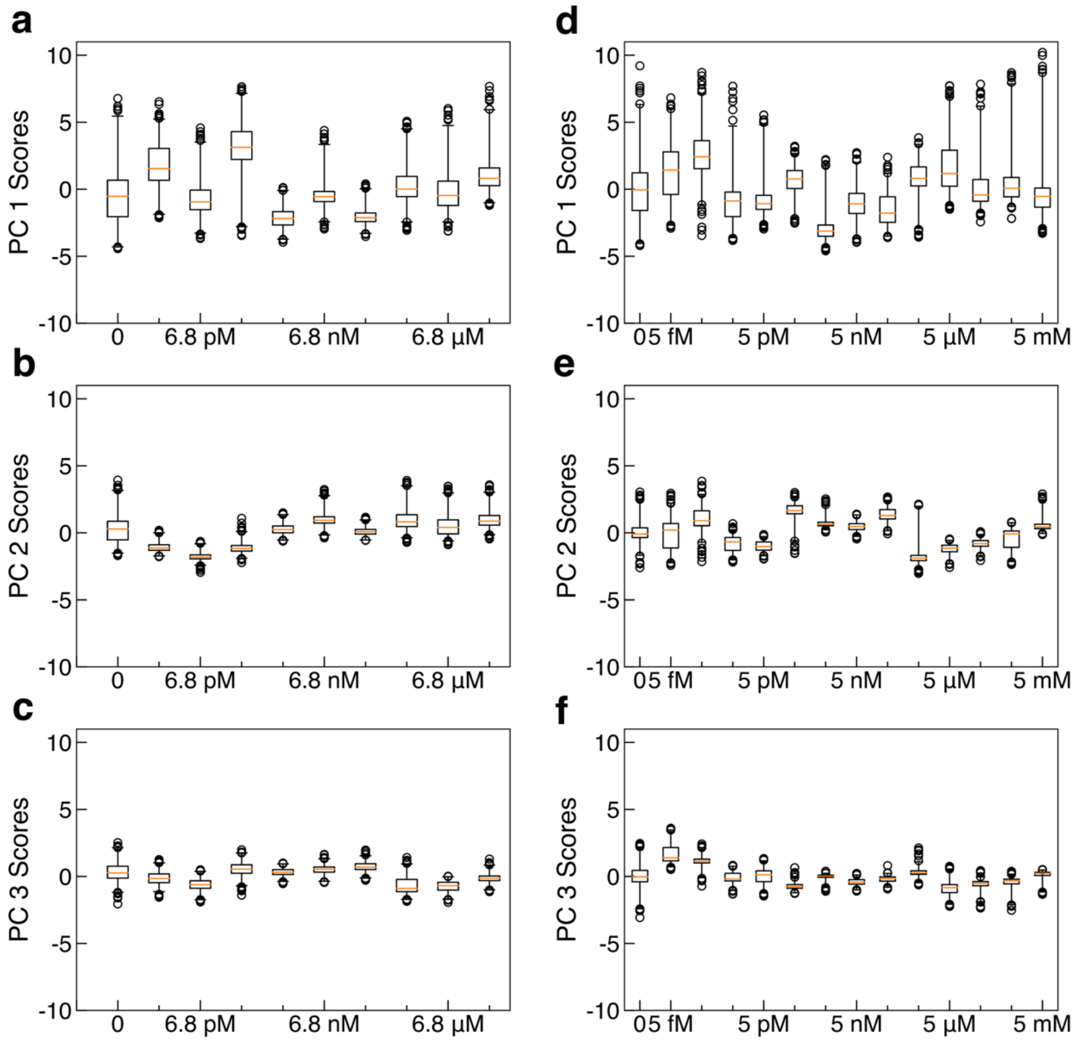

Fig. S4. Boxplot showing principal component 1, 2 and 3 scores for Cr^{6+} (**a, b, c**) and As^{3+} (**d, e, f**), respectively. Isolation forest was used for 1% outlier removal before plotting the data.

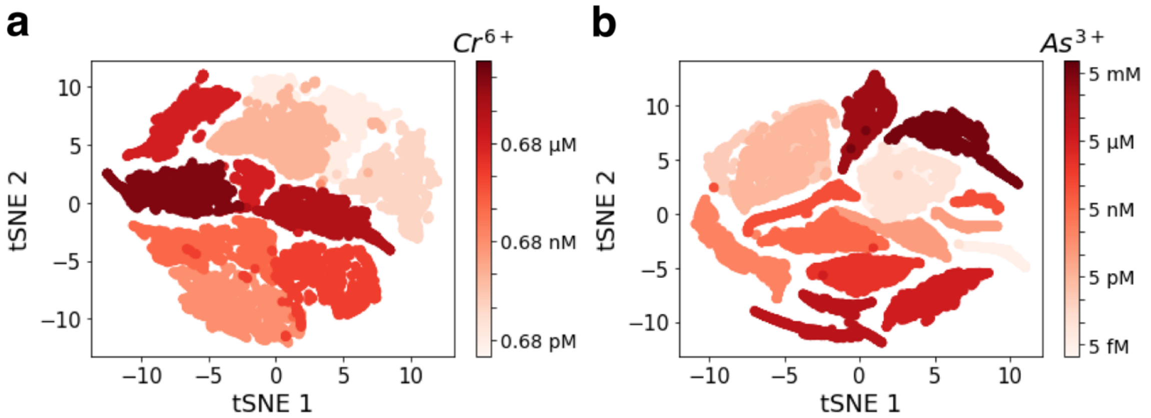

Fig. S5. t-distributed stochastic neighbor embedding cluster plot of Cr^{6+} (left) and As^{3+} (right) showing different metal exposure concentrations exhibit differential features when using unsupervised algorithms.

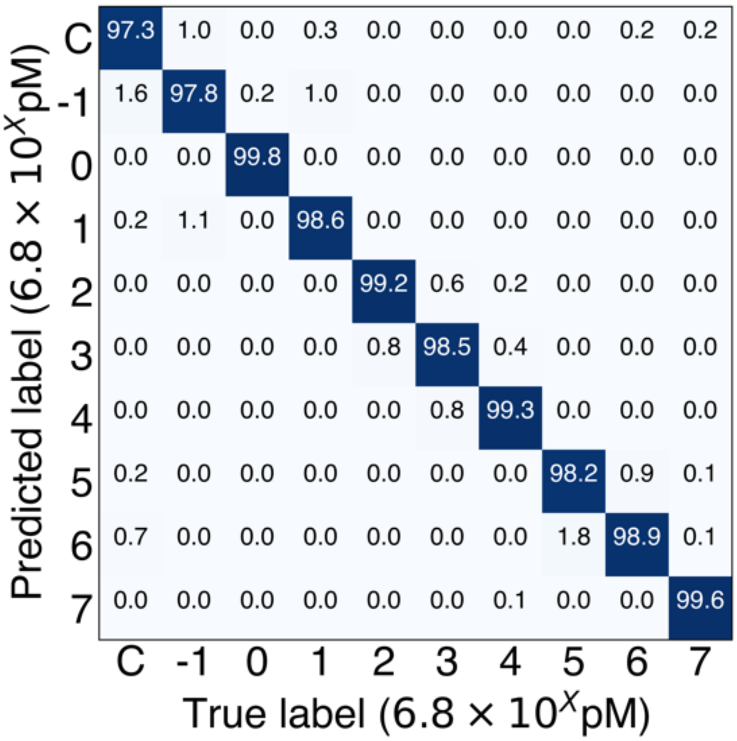

Fig. S6. Confusion matrices from 10-fold cross validation of SVM classification models for Cr^{6+} obtained from the training dataset.

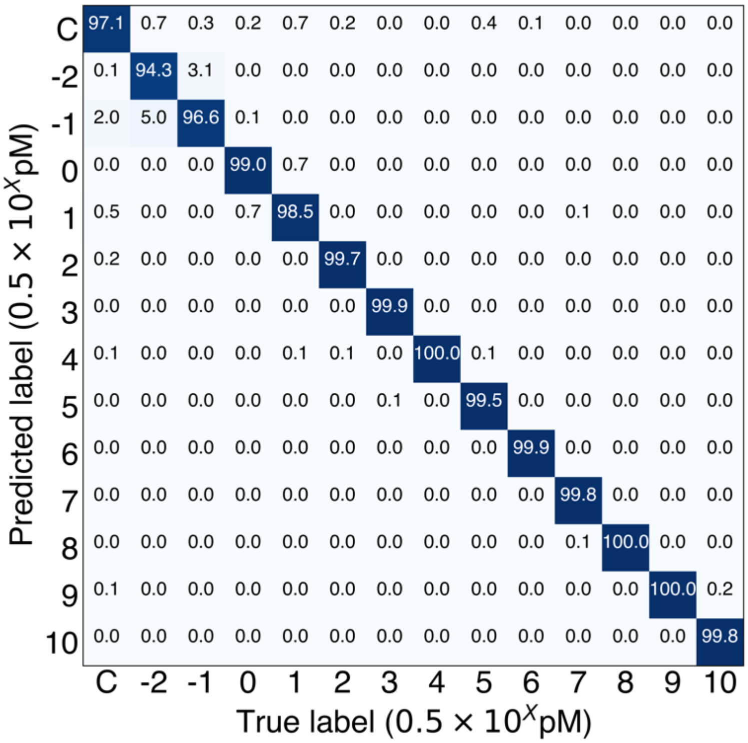

Fig. S7. Confusion matrices from 10-fold cross validation of SVM classification models for As^{3+} obtained from the training dataset.

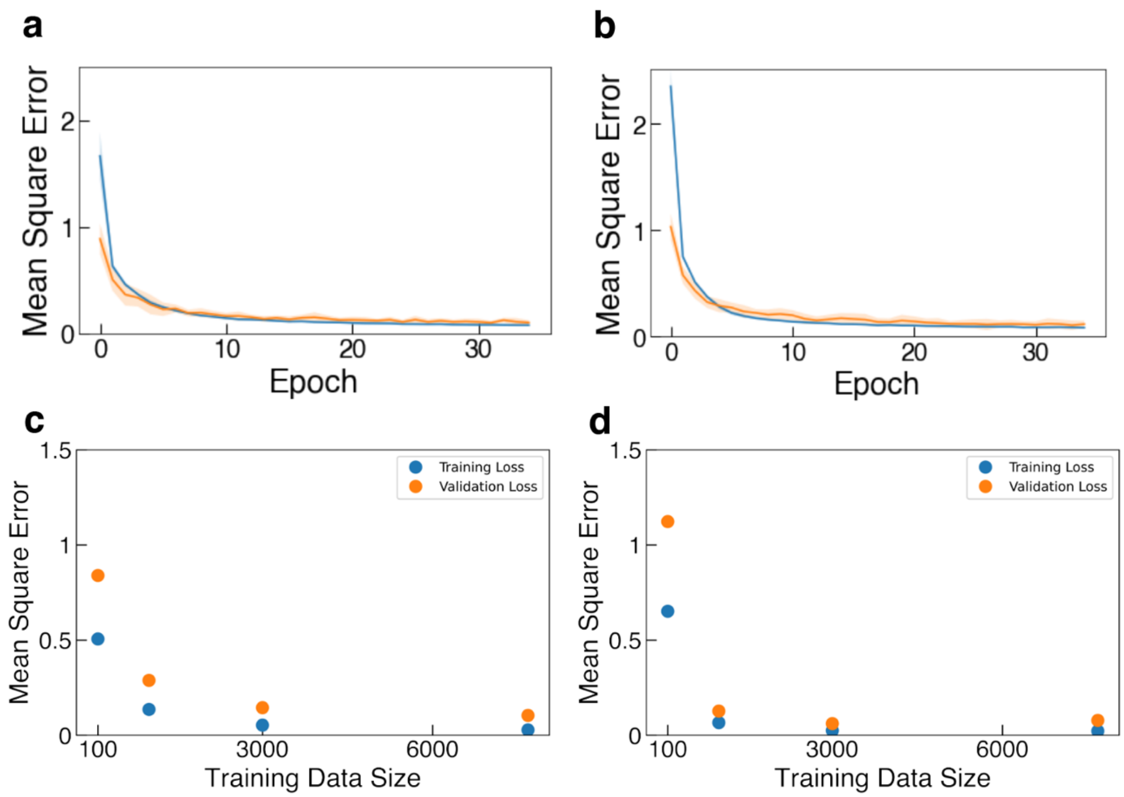

Fig. S8. Overlaid learning curve from 1D CNN regression model for **a** Cr^{6+} and **b** As^{3+}. The blue (orange) line is training (validation) mean loss, and the blue (orange) shading is training (validation) mean loss with standard deviation. The epoch size used in 1D CNN algorithm is 35. The blue dots represent training loss and the orange dots represents validation loss for **c** Cr^{6+} and **d** As^{3+} as a function of the training data size.

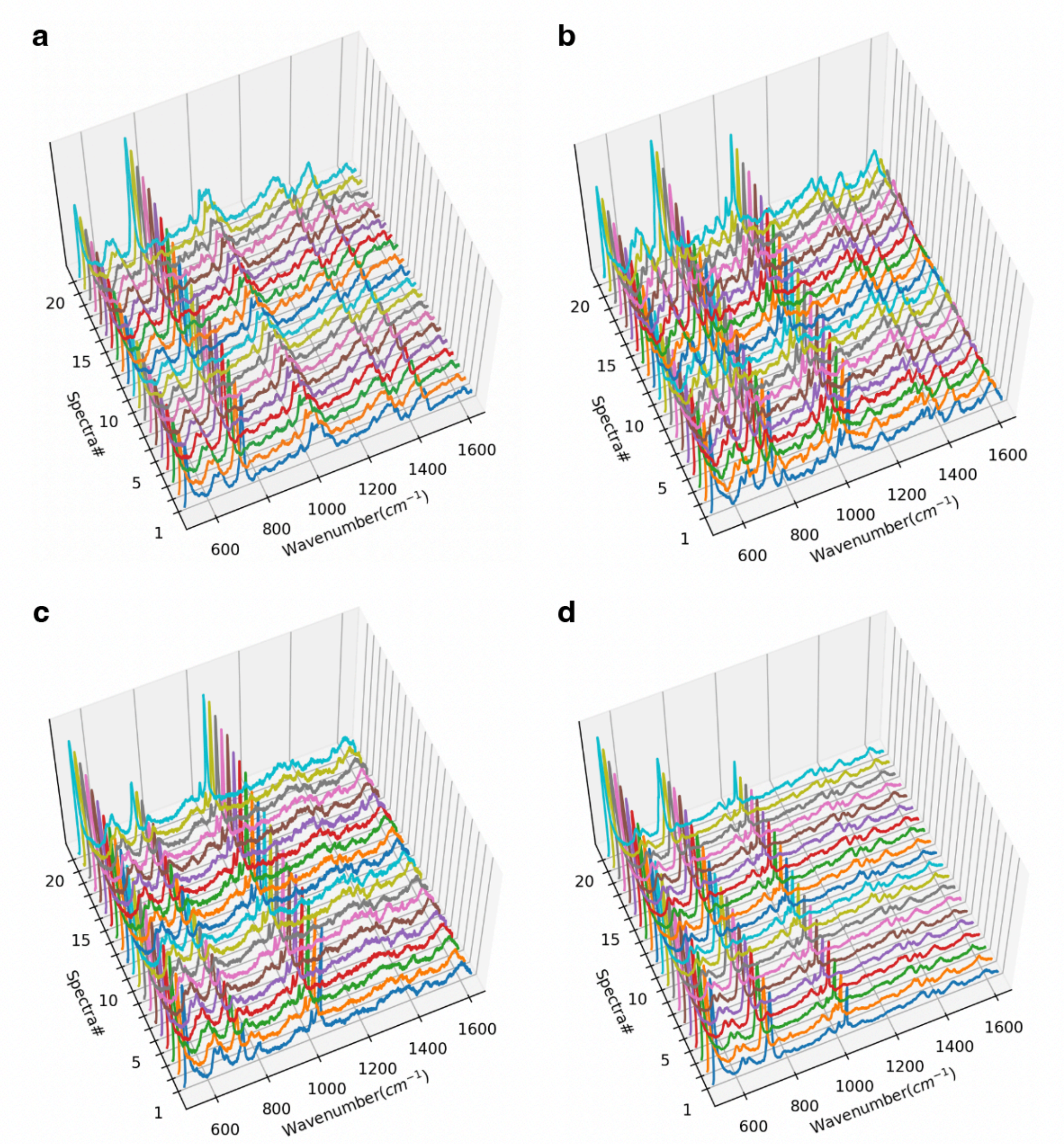

Fig. S9. Waterfall plots of 20 randomly chosen spectra at concentrations of **a.** 10x and **b.** 1000x LOD from Cr^{6+} (6.8 pM) and **c.** 10x and **d.** 1000x LOD from As^{3+} (0.5 pM) showing uniform signals across sensor surfaces.

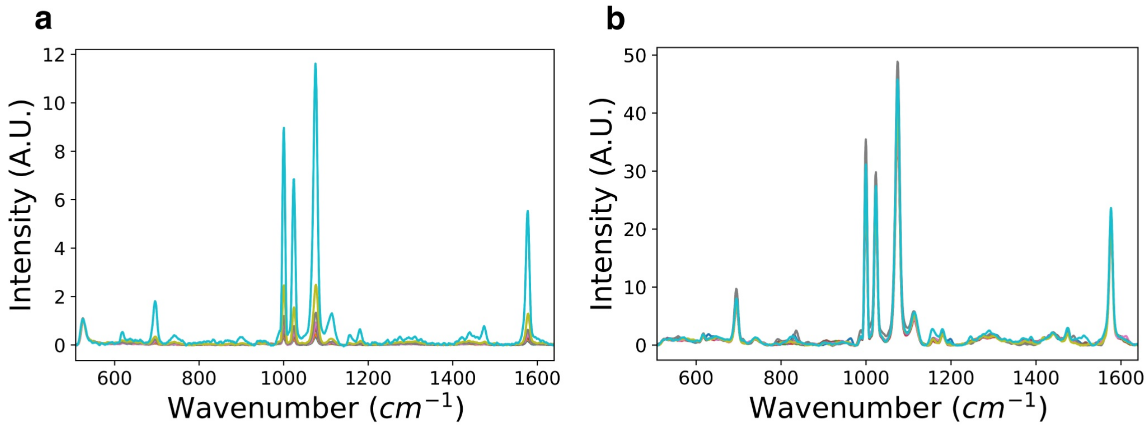

Fig. S10. Randomly selected spectra from SERS surfaces fabricated by a. Drop-casting Au nanoparticles and b. with EHD flow. Per convention, the silicon peak at 520 cm^{-1} is used as an internal standard and normalized to 1 for all spectra. The total SERS intensity is different in Fig. a and b due to different enhancement factors from the two types of surfaces.

Table S1. PC1, 2 and 3 loading peak relationship to metabolites for Cr^{6+} and As^{3+}.

	Cr^{6+} Features	Metabolite(s)	As^{3+} Features	Metabolite(s)
**PC1**	734 cm^{-1}	adenine[4–7]	734 cm^{-1}	adenine[4–7]
	1030 cm^{-1}	phenylalanine[8–10] adenosine[5]	724 cm^{-1}	hypoxanthine[11]
	1036 cm^{-1}	dAMP[5]	1030 cm^{-1}	phenylalanine[8,10] adenosine[5]
	1040 cm^{-1}	uracil[12], ATP[13], thymine[4]	1036 cm^{-1}	dAMP[5]
	1017 cm^{-1}	phenylalanine[14]	1000 cm^{-1}	phenylalanine[15,16]
**PC2**	1030 cm^{-1}	phenylalanine[8,10] adenosine[5]	734 cm^{-1}	adenine[4–7]
	724 cm^{-1}	hypoxanthine[11]	1269 cm^{-1}	lipid[17]
	1003 cm^{-1}	phenylalanine[18]	607 cm^{-1}	indole[19]
	1010 cm^{-1}	indole[20]	1000 cm^{-1}	phenylalanine[15]
	671 cm^{-1}	cysteine[17,21,22]	1506 cm^{-1}	indole[19]
**PC3**	734 cm^{-1}	adenine[4–7]	1030 cm^{-1}	phenylalanine[8,10] adenosine[5]
	1030 cm^{-1}	phenylalanine[8,10] adenosine[5]	734 cm^{-1}	adenine[4–7]
	1040 cm^{-1}	uracil[12], ATP[13], thymine[4]	652 cm^{-1}	guanine[23,24]
	671 cm^{-1}	cysteine[17,21,22]	671 cm^{-1}	cysteine[17,21,22]
	641 cm^{-1}	tyrosine[18]	1269 cm^{-1}	lipid[17]

Table S2. Calculated metrics of sensor performance from SVM confusion matrices in Figure 3.

Cr^{6+}	Sensitivity (%)	Specificity (%)	Accuracy (%)
0.68 pM	85.8	99.86	98.1
6.8 pM	100	100	100
68 pM	98	99.9	99.7
0.68 nM	97.2	99.9	99.6
6.8 nM	99.6	99.8	99.8
68 nM	99.2	99.9	99.8
0.68 µM	99.2	99.99	99.9

As^{3+}	Sensitivity (%)	Specificity (%)	Accuracy (%)
5 fM	93.9	99.5	98.8
50 fM	87.4	99.1	97.6
0.5 pM	99.1	99.9	99.8
5 pM	97.5	99.9	99.6
50 pM	97.6	100	99.7
0.5 nM	100	100	100
5 nM	100	100	100

$$Sensitivity = \frac{True\ Positive}{True\ Positives + False\ Negatives} \quad (1)$$

$$Specificity = \frac{True\ Negatives}{True\ Negatives + False\ Positives} \quad (2)$$

$$Accuracy = \frac{True\ Positive + True\ Negatives}{True\ Positives + True\ Negatives + False\ Positives + False\ Negatives} \quad (3)$$

Table S3. Priority pollutants analysis summary from 2019-20 Orange County Sanitation District resource protection division, pretreatment program annual report.[25] (As concentration of 2.52 μg/L is equivalent to 19.4 nM)

Monitoring location	Analysis	Total Average Concentration	UNIT	Flow (MGD)	Mass (lbs/day)
EFF-001	As	2.52	μg/L	101	2.12
EFF-001	Cd	0.02	μg/L	101	0.017
EFF-001	Cr	1.07	μg/L	101	0.898
EFF-001	Cu	4.92	μg/L	101	4.13
EFF-001	Hg	5.14	ng/L	101	0.004
EFF-001	Ni	7.75	μg/L	101	6.51
EFF-001	Pb	0.464	μg/L	101	0.39
EFF-001	Sb	1.32	μg/L	101	1.11
EFF-001	Se	5.81	μg/L	101	4.88
EFF-001	Zn	24.5	μg/L	101	20.6

Supplemental References

1. Biggs, K. B.; Camden, J. P.; Anker, J. N.; Duyne, R. P. V. Surface-Enhanced Raman Spectroscopy of Benzenethiol Adsorbed from the Gas Phase onto Silver Film over Nanosphere Surfaces: Determination of the Sticking Probability and Detection Limit Time. *J. Phys. Chem. A* **2009**, *113* (16), 4581–4586.
2. Madzharova, F.; Heiner, Z.; Kneipp, J. Surface-Enhanced Hyper Raman Spectra of Aromatic Thiols on Gold and Silver Nanoparticles. *J. Phys. Chem. C* **2020**, *124* (11), 6233–6241.
3. Samuel, A. Z.; Mukojima, R.; Horii, S.; Ando, M.; Egashira, S.; Nakashima, T.; Iwatsuki, M.; Takeyama, H. On Selecting a Suitable Spectral Matching Method for Automated Analytical Applications of Raman Spectroscopy. *ACS Omega* **2021**, *6* (3), 2060–2065.
4. Chan, T.-Y.; Liu, T.-Y.; Wang, K.-S.; Tsai, K.-T.; Chen, Z.-X.; Chang, Y.-C.; Tseng, Y.-Q.; Wang, C.-H.; Wang, J.-K.; Wang, Y.-L. SERS Detection of Biomolecules by Highly Sensitive and Reproducible Raman-Enhancing Nanoparticle Array. *Nanoscale Res. Lett.* **2017**, *12* (1), 344.
5. Bell, S. E. J.; Sirimuthu, N. M. S. Surface-Enhanced Raman Spectroscopy (SERS) for Sub-Micromolar Detection of DNA/RNA Mononucleotides. *J. Am. Chem. Soc.* **2006**, *128* (49), 15580–15581.
6. Madzharova, F.; Heiner, Z.; Gühlke, M.; Kneipp, J. Surface-Enhanced Hyper-Raman Spectra of Adenine, Guanine, Cytosine, Thymine, and Uracil. *J. Phys. Chem. C* **2016**, *120* (28), 15415–15423.
7. Yao, G.; Zhai, Z.; Zhong, J.; Huang, Q. DFT and SERS Study of ^{15} N Full-Labeled Adenine Adsorption on Silver and Gold Surfaces. *J. Phys. Chem. C* **2017**, *121* (18), 9869–9878.
8. Chan, J. W.; Taylor, D. S.; Zwerdling, T.; Lane, S. M.; Ihara, K.; Huser, T. Micro-Raman Spectroscopy Detects Individual Neoplastic and Normal Hematopoietic Cells. *Biophys. J.* **2006**, *90* (2), 648–656.
9. Cheng, W.-T.; Liu, M.-T.; Liu, H.-N.; Lin, S.-Y. Micro-Raman Spectroscopy Used to Identify and Grade Human Skin Pilomatrixoma. *Microsc. Res. Tech.* **2005**, *68* (2), 75–79.
10. Zhu, J.; Zhou, J.; Guo, J.; Cai, W.; Liu, B.; Wang, Z.; Sun, Z. Surface-Enhanced Raman Spectroscopy Investigation on Human Breast Cancer Cells. *Chem. Cent. J.* **2013**, *7*, 37.
11. Cui, L.; Chen, P.; Chen, S.; Yuan, Z.; Yu, C.; Ren, B.; Zhang, K. In Situ Study of the Antibacterial Activity and Mechanism of Action of Silver Nanoparticles by Surface-Enhanced Raman Spectroscopy. *Anal. Chem.* **2013**, *85* (11), 5436–5443.
12. Farquharson, S.; Smith, W. W.; Lee, V. Y.-H.; Elliott, S.; Sperry, J. F. Detection of Bioagent Signatures: A Comparison of Electrolytic and Metal-Doped Sol-Gel Surface-Enhanced Raman Media. In *Chemical and Biological Early Warning Monitoring for Water, Food, and Ground*; SPIE, 2002; Vol. 4575, pp 62–72.
13. Chen, T. T.; Kuo, C. S.; Chou, Y. C.; Liang, N. T. Surface-Enhanced Raman Scattering of Adenosine Triphosphate Molecules. *Langmuir* **1989**, *5* (4), 887–891.
14. Pan, J.; Shao, X.; Zhu, Y.; Dong, B.; Wang, Y.; Kang, X.; Chen, N.; Chen, Z.; Liu, S.; Xue, W. Surface-Enhanced Raman Spectroscopy before Radical Prostatectomy Predicts Biochemical Recurrence Better than CAPRA-S. *Int. J. Nanomedicine* **2019**, *Volume 14*, 431–440.
15. Ivleva, N. P.; Wagner, M.; Szkola, A.; Horn, H.; Niessner, R.; Haisch, C. Label-Free in Situ SERS Imaging of Biofilms. *J. Phys. Chem. B* **2010**, *114* (31), 10184–10194.
16. Cui, L.; Zhang, Y.-J.; Huang, W. E.; Zhang, B.-F.; Martin, F. L.; Li, J.-Y.; Zhang, K.-S.; Zhu, Y.-G. Surface-Enhanced Raman Spectroscopy for Identification of Heavy Metal Arsenic(V)-Mediated Enhancing Effect on Antibiotic Resistance. *Anal. Chem.* **2016**, *88* (6), 3164–3170.
17. Stone, N.; Kendall, C.; Smith, J.; Crow, P.; Barr, H. Raman Spectroscopy for Identification of Epithelial Cancers. *Faraday Discuss.* **2004**, *126*, 141–157; discussion 169-183.

18. Maquelin, K.; Kirschner, C.; Choo-Smith, L.-P.; van den Braak, N.; Endtz, H. P.; Naumann, D.; Puppels, G. J. Identification of Medically Relevant Microorganisms by Vibrational Spectroscopy. *J. Microbiol. Methods* **2002**, *51* (3), 255–271.
19. De Marchi, S.; Bodelón, G.; Vázquez-Iglesias, L.; Liz-Marzán, L. M.; Pérez-Juste, J.; Pastoriza-Santos, I. Surface-Enhanced Raman Scattering (SERS) Imaging of Bioactive Metabolites in Mixed Bacterial Populations. *Appl. Mater. Today* **2019**, *14*, 207–215.
20. Jayan, H.; Pu, H.; Sun, D.-W. Detection of Bioactive Metabolites in Escherichia Coli Cultures Using Surface-Enhanced Raman Spectroscopy. *Appl. Spectrosc.* **2022**, *76* (7), 812–822.
21. Yao, G.; Huang, Q. DFT and SERS Study of L-Cysteine Adsorption on the Surface of Gold Nanoparticles. *J. Phys. Chem. C* **2018**, *122* (27), 15241–15251.
22. Jing, C.; Fang, Y. Experimental (SERS) and Theoretical (DFT) Studies on the Adsorption Behaviors of l-Cysteine on Gold/Silver Nanoparticles. *Chem. Phys.* **2007**, *332* (1), 27–32.
23. Giese, B.; McNaughton, D. Density Functional Theoretical (DFT) and Surface-Enhanced Raman Spectroscopic Study of Guanine and Its Alkylated Derivatives. *Phys. Chem. Chem. Phys.* **2002**, *4* (20), 5171–5182.
24. Wang, W.; Hynninen, V.; Qiu, L.; Zhang, A.; Lemma, T.; Zhang, N.; Ge, H.; Toppari, J. J.; Hytönen, V. P.; Wang, J. Synergistic Enhancement via Plasmonic Nanoplate-Bacteria-Nanorod Supercrystals for Highly Efficient SERS Sensing of Food-Borne Bacteria. *Sens. Actuators B Chem.* **2017**, *239*, 515–525.
25. Orange County Sanitation District. 2019-20 Resource Protection Division Pretreatment Program Annual Report, 2020.
